# Monolithic Chip for High-throughput Blood Cell Depletion to Sort Rare Circulating Tumor Cells

**DOI:** 10.1038/s41598-017-11119-x

**Published:** 2017-09-07

**Authors:** Fabio Fachin, Philipp Spuhler, Joseph M. Martel-Foley, Jon F. Edd, Thomas A. Barber, John Walsh, Murat Karabacak, Vincent Pai, Melissa Yu, Kyle Smith, Henry Hwang, Jennifer Yang, Sahil Shah, Ruby Yarmush, Lecia V. Sequist, Shannon L. Stott, Shyamala Maheswaran, Daniel A. Haber, Ravi Kapur, Mehmet Toner

**Affiliations:** 1 BioMEMS Resource Center, Center for Engineering in Medicine and Surgical Services, Massachusetts General Hospital and Harvard Medical School, Boston, Massachusetts, 02114 USA; 2 Cancer Center, Massachusetts General Hospital, Boston, Massachusetts, 02114 USA; 3 Shriners Hospitals for Children, Boston, Massachusetts, 02114 USA

## Abstract

Circulating tumor cells (CTCs) are a treasure trove of information regarding the location, type and stage of cancer and are being pursued as both a diagnostic target and a means of guiding personalized treatment. Most isolation technologies utilize properties of the CTCs themselves such as surface antigens (e.g., epithelial cell adhesion molecule or EpCAM) or size to separate them from blood cell populations. We present an automated monolithic chip with 128 multiplexed deterministic lateral displacement devices containing ~1.5 million microfabricated features (12 µm–50 µm) used to first deplete red blood cells and platelets. The outputs from these devices are serially integrated with an inertial focusing system to line up all nucleated cells for multi-stage magnetophoresis to remove magnetically-labeled white blood cells. The monolithic CTC-iChip enables debulking of blood samples at 15–20 million cells per second while yielding an output of highly purified CTCs. We quantified the size and EpCAM expression of over 2,500 CTCs from 38 patient samples obtained from breast, prostate, lung cancers, and melanoma. The results show significant heterogeneity between and within single patients. Unbiased, rapid, and automated isolation of CTCs using monolithic CTC-iChip will enable the detailed measurement of their physicochemical and biological properties and their role in metastasis.

## Introduction

Circulating tumor cells (CTCs) are critical rare cell targets as they can be present in extremely low numbers (down to 1–10 per mL of whole blood) and have been shown to be a root cause of the majority of cancer related deaths. A great deal of research has delved into the detection, genomics and the implications of these cells in disease progression and monitoring^1–4^. From this rapidly expanding realm of research, CTCs have been explored for prognosis^5–13^, targeted therapies based upon detected genetic abnormalities^14, 15^, *ex-vivo* culture for personalized medicine^16^ and the investigation of the epithelial to mesenchymal transitions or EMT^17–19^. They have also been used for single cell genomic studies^20, 21^, monitoring response to treatments^20^ and led to the discovery of new therapeutic targets^22^.

Given the potential of CTCs both to advance our understanding of the biology of metastasis and in the management of cancer within patients, multiple isolation methods have been developed mostly based upon known surface markers and/or other physical property differences between cancer cells and blood cells. Positive selection technologies including CellSearch®^9^, the only FDA approved clinical test, utilize known surface markers (typically EpCAM) to isolate the CTCs from a blood sample. More recently, a microfluidic approach has been proposed for the isolation of CTCs using positive selection (CTC-chip)^23^. There are now a number of microfluidic technologies available including GEDI^24^, Magsweeper^25^, centrifugal lab-on-a-disk^26^ and the herringbone CTC-chip^27^ that sort CTCs using EpCAM and other surface antigens as target moieties. However, these surface molecules have been shown to dynamically vary in expression during disease states (e.g., EMT)^28, 29^, are not present on certain types of cancer cells such as those associated with melanoma, and patient CTCs typically express fewer copies of EpCAM than cancer cell lines typically used to validate new CTC technologies^30^. This suggests that tumor antigen based positive selection approaches might not be able to isolate the entire population of CTCs.

One strategy to overcome this pitfall is the use of size-based sorting technologies. Early work used microfilters^31^ while more recent studies involve the use of deterministic lateral displacement or DLD^32^, isolation by size of epithelial tumor cells or ISET^33^, and inertial focusing^34^. These technologies work on the presumption that CTCs are larger than typical blood cells, which is shown to be true for cancer cell lines but the limited amount of data with patient CTCs do not support this assumption^16, 17^. Furthermore, the CTC size statistics are biased by the type of isolation technology used^35–37^. Another approach that does not rely on any single protein based enrichment of CTCs is the use of high-definition CTC analysis developed by Kuhn and colleagues, where all nucleated cells are panned onto slides for staining and subsequent multi-marker immunofluorescent imaging to identify CTCs^37^. Although nucleated cells including CTCs are attached onto a dozen or so specially developed large slides for imaging along with millions of contaminating WBCs, and the cells are not alive as they are fixed for processing, this technique clearly supports the unbiased isolation of CTCs and useful for central laboratory type settings.

To overcome the shortcomings of the existing approaches, we engineered an inertial focusing-enhanced microfluidic system, the CTC-iChip, which allows for high-efficiency negative depletion of normal blood cells, leaving CTCs in solution where they can be individually selected and analyzed as single cells^21, 38^. The CTC-iChip combines hydrodynamic size-based separation of all nucleated cells (leukocytes and CTCs) away from red blood cells, platelets, and plasma, with subsequent inertial focusing of the nucleated cells onto a single streamline to achieve high-efficiency in-line magnetophoretic depletion of white blood cells (WBCs) that are tagged with magnetic beads in whole blood. This antigen-independent isolation of CTCs enables the characterization of CTCs with both epithelial and mesenchymal characteristics. Furthermore, the high quality of RNA purified from viable, untagged CTCs is particularly well suited for detailed transcriptome analysis. The CTC-iChip technology was successful but limited in its applicability due to long set up times, multiple manually interconnected chips and would have been difficult to implement within a clinical setting.

Here we present the culmination of microfluidic and process engineering efforts to develop a high-throughput monolithic CTC-iChip, which uses deterministic lateral displacement, inertial focusing, and magnetophoresis to deplete blood cells at 15–20 million cells per second to sort CTCs. The individual components previously manufactured using deep reactive ion silicon etching and PDMS (polydimethylsiloxane) soft lithography^21, 38^ have now been integrated on a single mass-produced plastic chip, vastly decreasing both technical requirements and hands on time and hence improving the accessibility of the technology. The CTCs are unbiasedly collected in solution, rather than attached on a surface or with beads attached to their membranes and they spend less than 8 seconds within the monolithic chip based upon average flow speeds in each device stage and their connecting channels. The characterization of both CTC size and EpCAM expression levels from cancer patients clearly shows the heterogeneity of the CTCs and unequivocally demonstrates the advantages of a negative selection based clinical CTC technology. Additionally, there are already a few examples of research taking advantage of the improved purity and throughput of this technology^39, 40^. This paper details the extensive characterization and rigorous testing of the limits of a technology that is already being utilized for clinical and scientific pursuits.

## Results and Discussion

**Figure 1:**
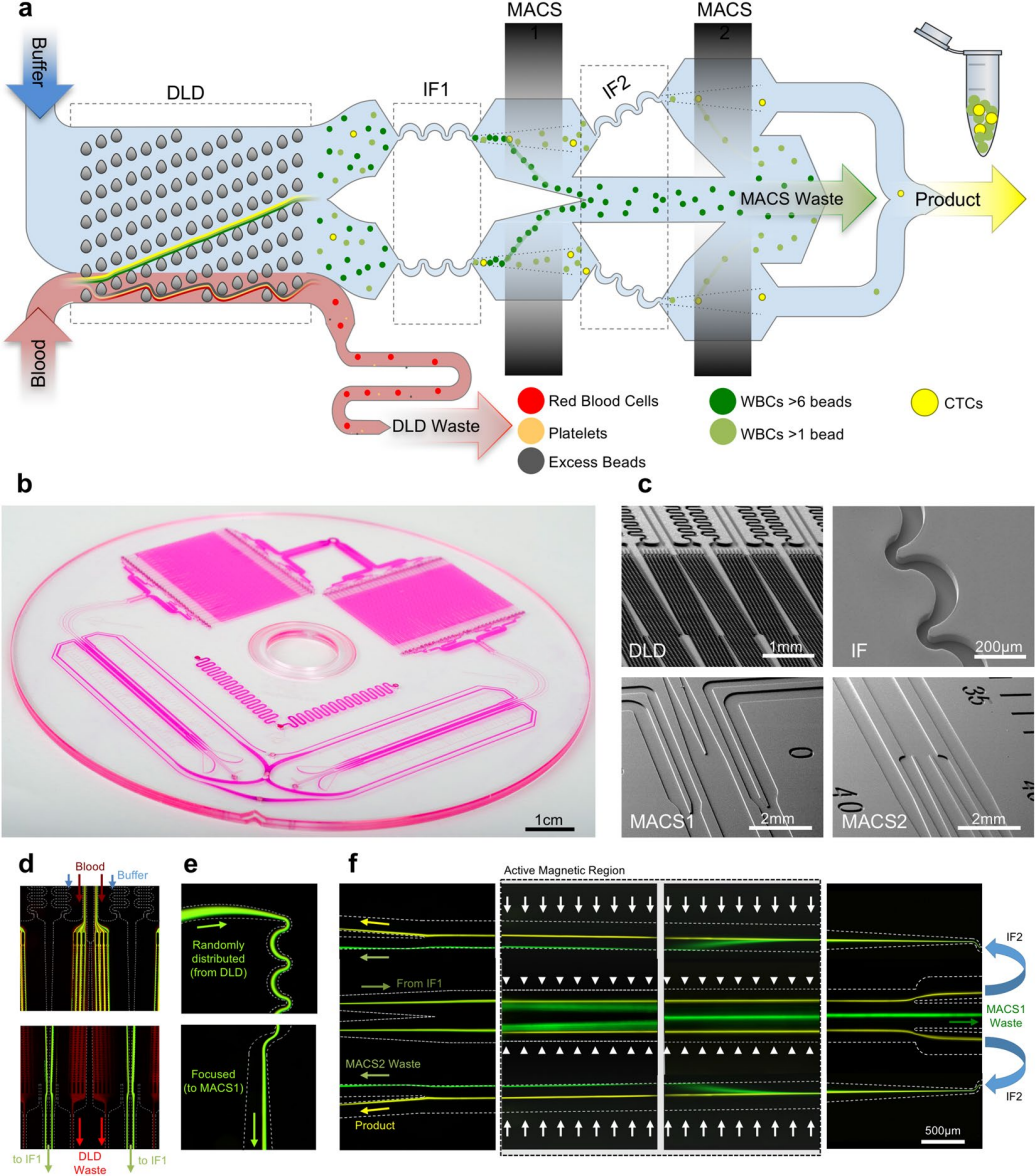
Device and Process Flow - (**a**) Linearized schematic of the monolithic chip and how cells are processed. (**b**) Photograph of a single chip filled with dye. (**c**) Scanning electron micrographs of important locations on the chip, top left: DLD structures, top right: Inertial focusing units, bottom left: MACS1 inlet merge and MACS 2 outlet, and bottom right MACS2 inlet and MACS1 outlet split. Blood pre-labeled with antibodies and magnetic beads targeting white blood cells and buffer enter the device and first go through a deterministic lateral displacement (DLD) device that separates nucleated cells from the red blood cells, platelets and unbound magnetics beads. These cells are then accelerated through an inertial focusing device (IF1) that causes the alignment of the cells without the addition of a sheath flow. These aligned cells merge into a single channel and then pass through a magnetic field where the high gradient forces magnetically tagged cells towards the center of the channel (MACS1). This channel then splits collecting highly tagged cells in the center waste and all remaining cells are refocused in a second inertial focusing device (IF2) and then enter a different region of the magnetic field where the gradient is higher leading to the removal of all labeled cells (MACS2). (**d**) Operational images of the different microfluidic components on the chip starting on the left with the DLD device where WBCs and CTCs are labeled green and RBCs are labeled red. The RBCs, WBCs and CTCs enter together along with a buffer co-flow (top). RBCs and smaller items pass straight through the array while the WBCs and CTCs are bumped across the array into a buffer co-flow and can be separated by splitting the flow streams at the outlet (bottom). (**e**) Next the WBCs and CTCs enter the inertial focusing channels where they begin as a diffuse stream (top) and exit in a tightly aligned fashion. (**f**) The last images on the righty show the flow of two inertial focusing channels (one half of the entire chip) (middle) flowing from left to right going through the low magnetic gradient region and achieving partial separation. The streams are split and the CTCs with few WBCs are refocused (not shown) and enter the second high gradient MACS region flowing from right to left. The now purified product of CTCs (yellow) is split from the WBCs stream at the outlet.

The monolithic microfluidic device incorporates three different microfluidic technologies for rapid and high-throughput negative selection of circulating tumor cells that is completely controlled using on-chip fluidic resistors and a single pressure source. The device consists of an injection molded fluidic layer that contains all the microfluidic features, which is then thermally bonded to an injection molded lid-layer containing molded through-holes for micro-macro interfacing. The microfluidic disks are made of medical-grade cyclic olefin copolymer (COC) and are manufactured using direct laser writing mastering in combination with variothermal (localized time-dependent temperature control) injection-compression molding. A molded COC on-chip-cassette that is laser welded to the lid layer enables connectivity with the instrumentation and allows interfacing between the different microfluidic subcomponents. Images of these different layers, how the fluid moves between these layers and the apparatus created for operating the chip can be found in Supplementary Figure 1. The overall symmetrically parallelized chip architecture includes the integration of the following five microfluidic stages represented schematically in Fig. 1a: deterministic lateral displacement (DLD), inertial focusing stage 1 (IF1), magnetically-activated cell sorting stage 1 (MACS1), IF2 and MACS2. An overall image of an integrated chip is shown in Fig. 1b with four scanning electron micrographs of key fluidic features from these devices shown in Fig. 1c.

Tumor cell isolation is achieved by inputting whole blood that is pre-labeled with magnetic beads targeting white blood cells via CD45, CD16 and CD66b surface antigens, as well as a running buffer. These two fluids are equally distributed between 128 DLD arrays as side-by-side co-flows. The DLD arrays are designed similarly to those used in our previous work^21, 38^ with adjustments described in Supplementary Figure 2. As such, using a designed cell diameter cutoff of 3.8 µm, nucleated cells are bumped into the buffer co-flow while red blood cells (RBCs), platelets, plasma proteins and excess labeling materials are removed as shown in Fig. 1d on the left. The average fluid speed through these arrays is 5 mm/s leading to a throughput of ~15 million cells/second. The DLD arrays separate an average of 70% of the nucleated cells from the original blood sample to the following inertial focusing and magnetophoresis portions of the chip. The majority of lost nucleated cells are small (lymphocytes) or highly deformable leukocytes (neutrophils), as shown in Supplementary Figure 3, in addition to any CTCs that are of similar size and deformability (<~5 µm). Distinct from our previous works where control of the DLD waste flow rate was achieved via the use of an external syringe pump, the integrated chip architecture presented utilizes an on-chip hydraulic resistor to control the relative flow exiting through the DLD waste channels versus the fluid that is allowed through the IF and MACS devices. To enable this on-chip DLD waste control, the individual DLD waste streams are first redirected via lid layer through-holes to two reservoirs within the on-chip cassette; from here, the DLD waste flow is then redirected towards two other lid-layer through-holes that feed microfluidic channels referred to as “DLD waste resistors” with precisely engineered hydraulic resistance. These are used to control the balance of flow of the DLD waste and the fractions passing through IF and MACS stages, 78% and 22% of volumetric flow rate respectively, matching the designed cross-sectional area collection percentages for the DLD arrays (~2/10 fluidic lanes). Collection of two fluidic lanes to DLD product mitigates manufacturing imperfection and variability of sample biology and cell density (See Supplementary Information for more details about these design simulations/calculations).

Within each mirrored disk-half, a network of progressively widening branching structures combines the nucleated cell streams from the DLD arrays and converges them towards a first pair of sheathless alignment inertial focusing channels (IF1; see Fig. 1e). These IF channels align cells using a balance of three purely hydrodynamic forces: a shear gradient lift force, a wall interaction force and a drag force caused by a secondary flow known as Dean flow^41^. Once the cells are aligned, each pair of inertial focusing channels merge and expand into a 1 mm wide channel, thus decreasing the average speed of the cells as they enter the first magnetic field region (MACS1). This region has a mean magnetic flux gradient magnitude of 200 T/m, which causes magnetophoretic separation of magnetic bead-labeled cells to the center of the channel and into a waste channel (MACS waste). By moving these cells towards the center of the channel rather than towards the walls, the possibility of plaque formation (cells and magnetic beads) at the low shear zones near the walls is greatly diminished. The sensitivity of this first stage enables the separation of cells labeled with greater than ~6 magnetic beads (1 µm diameter Dynal MyOne superparamagnetic beads). The remaining cells enter a second set of inertial focusing channels (IF2) and are then passed through a second magnetic region (MACS2) with a mean magnetic flux gradient magnitude of 425 T/m, which removes cells that are labeled with at least 1 magnetic bead and sends them to MACS waste. Un-labeled cells will not be affected by either MACS stage, and will proceed towards the product output. Fluorescent streak images of the operation of both MACS stages can be found in Fig. 1f. The MACS wastes from the two disk-halves are combined on the cassette layer via a curvilinear reservoir, which are then combined and exit the system via a single port. The product streams from the two disk-halves are pooled at the chip level and exit the system via a single cassette-layer port located between the DLD waste and MACS waste ports.

As all the fluidics are integrated and controlled solely by the design and applied pressure at the blood and buffer inlets, understanding the cascading effects of device dimensions are critical to successful and reliable operation. To this end extensive analysis of the fluidic resistance network was done in order to understand both uniform depth errors and local dimensional errors. These analyses are discussed in greater detail in the Supplementary Information (Supplementary Figures 4 and 5). Device fabrication processes and resulting device dimensions were monitored at the batch level and held to strict tolerances based upon these analyses.

Baseline operational performance was next rigorously characterized across 44 spiked cell line experiments using 11 different cell lines (SkMel28, H1650, H1975, H3122, LNCAP, PC3, PC3-9, VCAP, MB231, MCF-7, SkBR), the device achieved a median recovery of spiked cells of 99.5% with a median WBC carryover of 445 WBCs per mL of input blood. The relative yield, defined as the number of spiked cells found in the product divided by the total number found in both the product and the two MACS wastes, and purity of healthy donor samples are presented in Supplementary Figure 6 for different spiked cells lines with an average spike concentration of 425 cells/mL of input blood and a range from 19 to 5000 cells/mL. The yield was maintained across the tested range of input cell concentrations (Individual run concentrations given in Supplementary Table 1). The purity is on par with other advanced rare cell isolation technologies^42^ but lower than single cell picking or fluorescence based sorting^43^. Note that the one low yield point for PC3s was from a chip that clogged and not normal operation.

The CTC-iChip was then utilized for the processing of 38 patient blood samples using a standard protocol described in the Methods. Each patient sample was run within 6 hours of the sample being collected with an average sample processing time including setup and labeling all the way to enumeration of about 6–7 hours. The CTC-iChip product was concentrated 50x, from 5.5 mL to ~110 µL, using a microfluidic concentrator (~95% recovery of WBCs and cell lines)^44^ at a rate of 0.5 mL/min, then stained and analyzed with an imaging flow cytometer (Amnis ImageStreamX Mark II) to quantify CTC size and surface antigen expression, a process which took typically 1 hour. A CTC is identified based on presence of a nucleus (DRAQ5), CD45/CD66/CD16 negativity and EpCAM (Lung, Prostate or Breast CTCs) or CD146 and NG2 positivity (Melanoma CTCs). Additionally, qualitative scoring criteria are used to exclude cells as CTCs. These criteria include inspection of cell morphology indicating presence of a cell nucleus and an intact membrane in the bright field image and continuous surface membrane staining of EpCAM or CD146 and NG2. Overall, 2561 putative CTCs were collected and analyzed from four different patient populations with mostly stage 4 cancer (Supplementary Table 2 contains patient details): Melanoma (2 positive samples – total of 33 CTCs), Lung (9 positive samples – total of 92 CTCs), Prostate (2 positive samples – total of 913 CTCs) and Breast (26 positive samples – total of 1523 CTCs). Following the identification of CTCs, the cells are analyzed for size and mean membrane expression.

**Figure 2:**
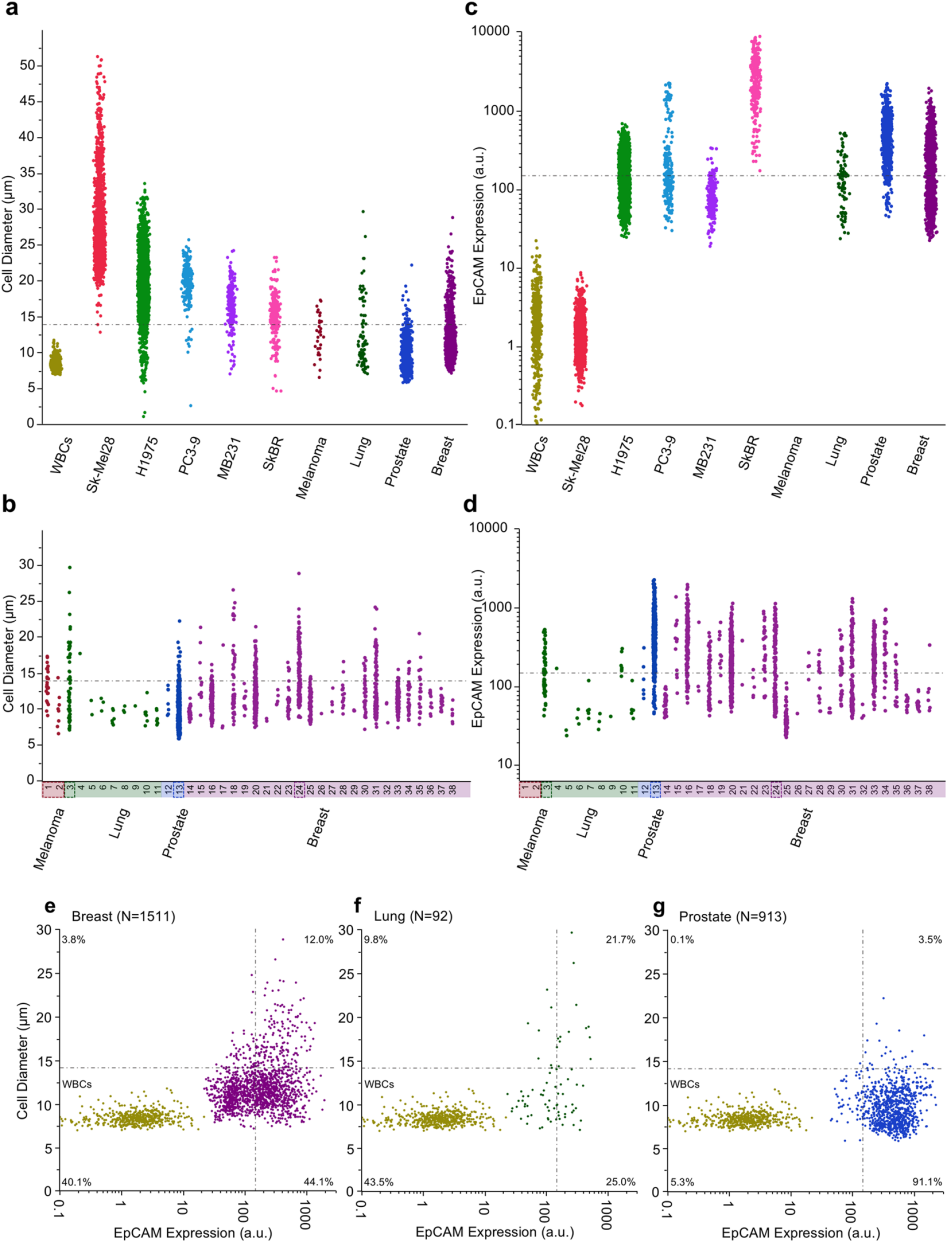
Size and Expression Comparison of Cell Lines and Patient CTCs – The size distributions of the CTCs, cell lines - Melanoma (SK-Mel28 – 2500 cells), Lung (H1975 – 5514 cells), Prostate (PC3-9 – 225 cells) and Breast (MB231 – 201 cells, SkBR – 295 cells) and healthy donor white blood cells obtained via running the CTC-iChip without magnetic beads (1 healthy donor sample – 620 cells) categorized by (**a**) cell population and (**b**) patient. Cell lines were significantly larger than their corollary CTC populations (All p < 0.001 Wilcoxon Rank Sum Test). The horizontal line at 13.9 µm indicates the size above which there are only 0.1% of WBCs. (**c**,**d**) The EpCAM expression distributions of the CTCs, cell lines and white blood cells categorized by cell population (left) and patient (right). Note that melanoma patient samples were excluded from the EpCAM measurements as they are generally accepted to not express EpCAM. Cell lines were significantly different in expression than their corollary CTC populations (All p < 0.001 Wilcoxon Rank Sum Test). The horizontal line at 147 a.u. indicates the estimated CellSearch® threshold for capture. Flow cytometry images from individual patients in four distinct categories emphasizing the heterogeneity of cells even within a single patient are given in Supplementary Figure 9. The patients from which these cells were sampled from are boxed with dashed lines. Size vs EpCAM – A comparative presentation of individual cell expression and size for patient CTCs with WBCs presented as a reference point for all (**e**) breast cancer, (**f**) lung cancer and (**g**) prostate cancer patient CTCs. NOTE: Here the EpCAM expression is the mean expression intensity rather than an absolute number. The two thresholds defined for size (13.9 µm) and EpCAM expression (147 a.u.) are represented by the horizontal and vertical dotted and dashed lines respectively. The quadrants are labeled with the percentage of patient CTCs in each.

In order to show that monolithic CTC-iChip isolates CTCs independent of size, we measured the size of CTCs after isolation from cancer patients. The size distributions of the CTCs are presented in Fig. 2a with the cumulative populations shown alongside the size distributions of various cancer cell lines and white blood cells. As expected, cell lines were found to be significantly larger than their corollary CTC populations (All p < 0.001 Wilcoxon Rank Sum Test). More importantly, a highly significant overlap of CTC sizes with the size distribution of the white blood cell population was also found. To aid in seeing this overlap a dot-dashed line is given at 13.9 µm given as the quantile above which there are only 0.1% of WBCs. The percentage of CTCs found below this threshold are 84.0% for breast patients, 68.5% for lung patients, 96.4% for prostate patients and 63.6% for melanoma patients. Supplementary Figure 7a shows the expected CTC yield of a purely size based technology as a function of this threshold and the CTC populations seen here. No selective loss of larger cells was found when running a pure population of H1975 cells through the CTC-iChip where the input population had a mean diameter of 19.5 µm (5514 cells measured) and the output from the CTC-iChip had a mean diameter of 20.0 µm (1652 cells measured). In Fig. 2b, the same size based measurements are presented but separated by patient sample clearly showing the variability in size between (largest CTC – 29.67 µm, patient sample 3, and smallest CTC – 5.86 µm, patient sample 13) and within a single patient sample (largest single patient CTC range 7.09 µm to 29.67 µm, patient sample number 3) and that some patients have only smaller CTCs (19/38 samples contained CTCs all smaller than the 13.9 µm threshold).

Direct comparisons with the size distributions presented in the literature are difficult due to dissimilar isolation procedures; however, the presented results (CTC diameters ranging from 5.5 µm to 27 µm) are generally in the same range as the reported values. Lazar *et al*. found that prostate CTCs were significantly smaller than LNCAP cells isolated utilizing RBC lysis and plating, 10.64 µm versus 13.49 µm respectively^45^. Park *et al* found that castration resistant prostate CTCs isolated using CellSearch® were significantly smaller than those from cultured cell lines^46^. Allard *et al* noticed that there was a large range of CTC sizes from 4–30 µm in diameter isolated using CellSearch® from patients with various malignant carcinomas^37^. Coumans *et al* isolated cells using different filter types and pore sizes finding that on average patient CTCs (breast = 13.1 µm, prostate = 10.0 µm and colorectal 11.0 µm) were larger than white blood cells^47^. Using an inertial focusing device to isolate CTCs from lysed blood, Hou *et al* found an average CTC size from 20 metastatic lung cancer patients of 16 µm and large size ranges were even found within single patients (10–22.5 µm in diameter)^42^. An important consideration in the sizing is our image analysis method, which determined the size of cells from fluorescent images of single cells. This method overestimates cell size by as much as 20%, however, this overestimation diminished with decreasing fluorescence intensity (See Supplementary Figure 8). Note that the data presented does not account for this discrepancy.

We also tested the ability of monolithic CTC-iChip to isolate CTCs independent of EpCAM expression in cancer patients. The EpCAM expression of the CTCs, cell lines and white blood cells are presented in Fig. 2c and d in terms of cumulative populations and individual patients, respectively. As expected both WBCs and melanoma cell lines expressed minimal EpCAM, likely attributed to noise or non-specific binding (WBC mean = 1.98 a.u., SkMel-28 mean = 1.40 a.u.). The other cell lines increased in average EpCAM expression in the following order MB231 at 93.8 a.u., H1975 at 182.9 a.u., PC3 at 369.7 a.u. and SkBR at 2980 a.u. Each patient group of CTCs was significantly different (p < 0.001 using Wilcoxon Rank Sum test) in expression of EpCAM than their cell line counterparts (Breast mean = 247.8 a.u., Lung mean = 163.0 a.u. and Prostate mean = 525.3 a.u.). For means of comparing these distributions to CTCs found via CellSearch® a threshold is defined as the equivalent EpCAM expression in a.u. corresponding to CellSearch® recovery yields found in the literature (SkBR – 85–99% yield^37^ ~1100–411 a.u., PC3 – 40% yield^48^ ~117 a.u. and MB231 – 12% yield^49^ ~147 a.u.). A compiled table of CellSearch® yields of numerous cell lines can be found in Supplementary Table 3. Using the median (147 a.u.) of these threshold values, 44.0% of breast CTCs, 53.3% of lung CTCs and 5.4% of prostate CTCs were found to be below the threshold. From an individual patient perspective, one third of the positive samples (13/38) contained only cells below the threshold (6 out of 9 lung samples, 0 out of 2 prostate sample and 7 out of 25 breast samples). Supplementary Figure 7b shows the expected CTC yield of an EpCAM positive selection technology as a function of this threshold and the CTC populations seen here. Example imaging flow cytometry images of a variety of patient CTCs from each disease type are presented in Supplementary Figure 9. The dashed boxes in Fig. 2 highlight the patients from which the CTC images were taken. Figure 2e, f and g summarize both the size and EpCAM expression of each individual CTC and the percentages of CTCs found in each of the 4 quadrants delineated by the size and EpCAM expression thresholds with the white blood cell population also plotted for comparative purposes. These interesting findings showed that proportions of EpCAM negative CTCs varied between disease type.

With respect to EpCAM expression, quantitative measurements are relatively limited in the literature after CTC isolation. The majority of available information surrounds the ability of EpCAM dependent isolation technologies and their efficiency isolating cell lines. Most sources take the pathological approach of defining expression as high and low rather than quantitatively. It should be noted that variation in EpCAM expression has been linked to the different types of breast cancer^50^. EpCAM measurements of cell lines are more common and the distributions found here qualitatively match conventional flow cytometry results from similar cell lines with data available in the literature^21^.

**Figure 3:**
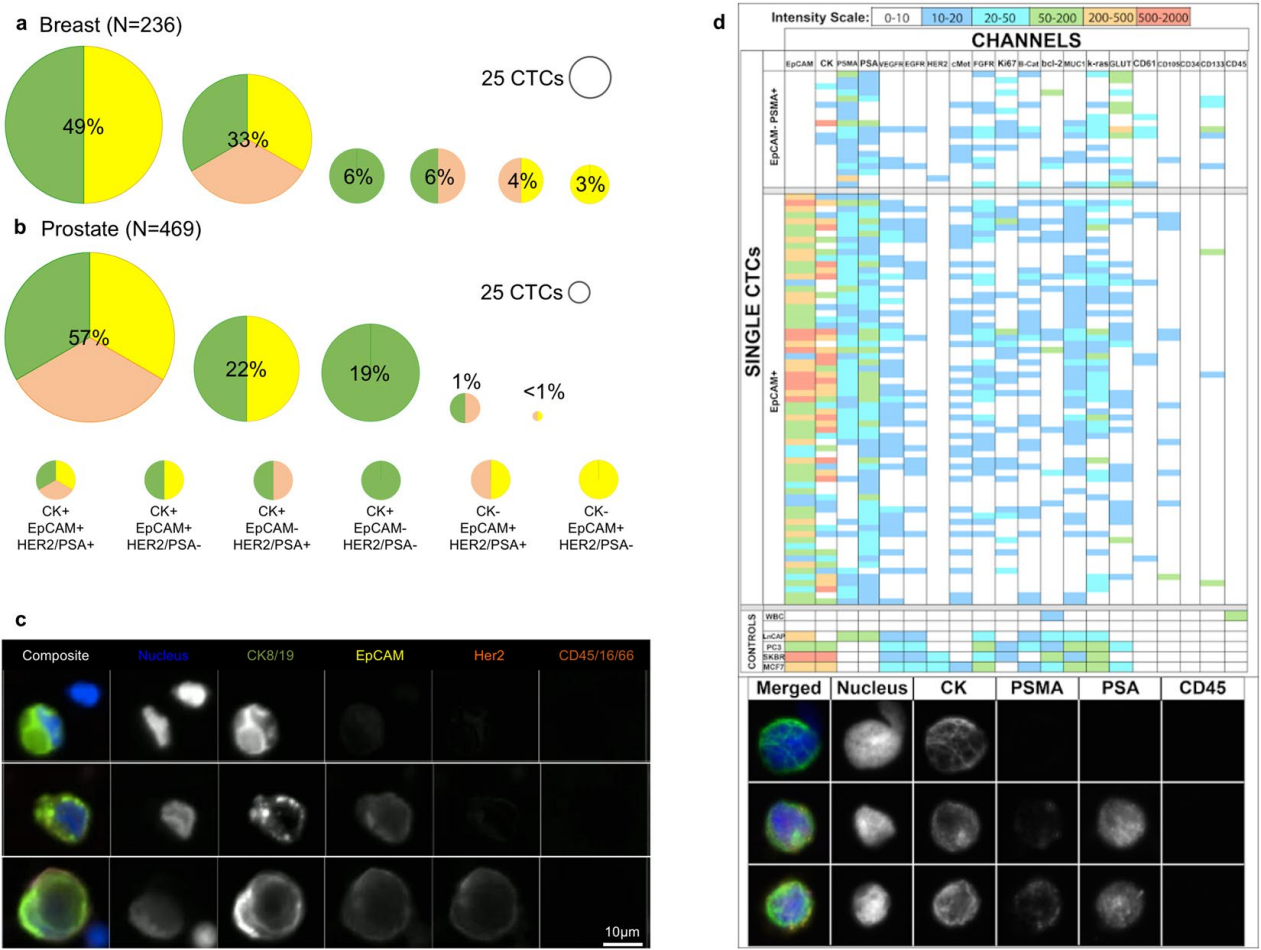
Heterogeneity of Cells Collected – Proportions of collected CTCs in different bins based upon positivity of surface and intracellular markers from (**a**) breast cancer patients and (**b**) prostate cancer patients. (**c**) Example images of CTCs from a single breast cancer patient. (**d**) Top panel: Table view of single CTCs identified via their positivity for PSMA and/or EpCAM (top rows) and control cell lines or white blood cells (bottom rows, represents median). Each row represents a single nucleated event and each column represent a channel detecting specified antigen. Color scale is denoted in the bottom, with white representing signal below S/N threshold set based upon controls. Bottom panel: a parallel slide based analysis of the same sample confirmed positive cells and their immunostaining for tumor marker. We detected 45 CTCs from slide-based analysis and 86 from mass cytometry based analysis from a total of 12 mL blood. (CK: cytokeratin, PSMA: prostate specific membrane antigen, PSA: prostate specific antigen).

To further demonstrate the value of antigen independent isolation of CTCs, we also investigated other cancer markers (cytokeratin - CK, human epidermal grown factor receptor 2 - HER2 and prostate specific antigen - PSA). Isolated cells from the monolithic CTC-iChip, were plated, fixed and stained, finding broad variation in cytokeratin expression as well as other typical disease-specific cancer cell markers. Figure 3a presents the proportions of CTCs found from a separate pool of 7 breast cancer patients and 3b prostate cancer patients indicating cells of interest that are in varying classifications based upon cytokeratin, EpCAM and either HER2 or PSA expression (See Supplementary Table 4 for specific scoring criteria and discussion). While most breast CTCs were found to be CK positive there were a few CK negative cells (7% of CTCs 17/236 cells) recovered using the CTC-iChip. Less than 1% of prostate CTCs found were cytokeratin negative (1/469 cells). EpCAM negative CTCs were also found in significant amounts across disease types, 12% of breast patients and 20% of prostate patients. CTCs negative for EpCAM and either HER2 or PSA were also identified at rates of 6% and 19%, respectively. Images of exemplar cells in these different classifications are shown in Fig. 3c.

Mass cytometry analysis was then performed on a single castration resistant prostate cancer patient sample. Using this technique, 86 distinct individual CTCs were analyzed for their expression of 20 individual cell markers presented in Fig. 3d. CTCs were defined here as EpCAM and/or PSMA positive events. Overall, 22% (19/86) of CTCs were PSMA+ and EpCAM−, 20% (17/86) of CTCs were EpCAM+ and PSMA− and 58% (50/86) of CTCs were EpCAM+ and PSMA+. The EpCAM expression, PSMA, PSA, and cytokeratin levels ranged across 3 orders of magnitude in these cells again highlighting the heterogeneity of the CTC populations that can exist within a single patient. The other remaining nucleated events (EpCAM/NG2−/CD146−) in the CTC-iChip product were analyzed using a deconvolution assay to determine the relative populations of leukocytes (DRAQ5+/CD45+/ CD16+/Glycophorin A−/Concanavalin A−/CD41−), erythroblasts (DRAQ5+/Glycophorin A+/CD45−/ CD16−/Concanavalin A−/CD41−), bare nuclei (DRAQ5+/Concanavalin A+/Glycophorin A−/CD45−/CD16−/CD41−), and megakaryocytes (DRAQ5+/CD41+/Concanavalin A−/Glycophorin A−/CD45−/CD16−). Nucleated cells with satellite platelets, which are also high expressers of CD41, were differentiated from megakaryocytes by their bright field morphology and the continuous membrane staining (megakaryocytes) relative to punctate staining of satellite platelets on nucleated events. The deconvolution assay successfully accounted for >90% of contaminant nucleated events in the product from 4 healthy donor samples and 4 melanoma patient donor samples, and the order of most abundant to least abundant nucleated events was found to be bare nuclei (median = 46%), leukocytes (median = 28%), erythroblasts (median = 16%), and megakaryocytes (median = 1.7%). The DAPI+ only events were also seen in the multispectral image analysis as seen in Fig. 3c and those events likely fall into one of these categories as well as potentially being CTCs undergoing phenotypical changes (i.e. epithelial-mesenchymal transition). Example images of each of these cell types and an explanation of the methods used to distinguish them can be found in Supplementary Figures 10 and 11 with complementary mass cytometry data for similar remaining nucleated events shown in Supplementary Figures 12 and 13. Other negative selection style assays targeting CTCs have found many more contaminating nucleated events^51, 52^. One patient had a significantly higher number of nucleated RBCs (132 NRBCs/mL), which has been associated with poor prognoses^53, 54^. The mass cytometry detailed the ranges of expression levels of numerous different markers and showed that no one uniformly expressed between all the single patient’s CTCs. This type of heterogeneity is corroborated by attempts to utilize cocktails of antibodies for improved performance of positive selection methodologies^55^ in addition to our previous CTC-iChip results^21^.

In summary, the monolithic CTC-iChip technology is an automated, fully integrated, plastic chip to isolate CTCs independent of epitope and size. This study finds that neither CTC size nor EpCAM expression can maximize isolation efficiency as many CTCs found were small and expressed lower levels of EpCAM. More importantly, both parameters were found to be significantly dependent on the individual patient and widely variable within a single patient. These results will guide the design of future CTC isolation and diagnostic strategies based on negative depletion of blood cells as well as further our biological understanding of the role of CTCs in metastasis.

### Human subjects

All experimental protocols using patient samples were reviewed and approved by the Dana-Farber Cancer Institute IRB, in accordance with the applicable Federal guidelines and regulations. Dana–Farber/Harvard Cancer Center IRB-approved protocol (Protocol No: 05-300) was used to obtain consent from all patients and healthy donors. The expiration date for the protocol is 03/01/2018.

## Methods

### Device Design, Fabrication and Optimization

Individual devices were designed based upon previous design iterations using separate chips coupled through tubings and manifolds^21, 38^. Detailed design considerations specific to the integrated monolithic chip will be presented here. In general, the design was created to work with the fabrication process that included direct laser writing of the master followed by injection-compression molding. A consequence of the direct laser writing process was that the stepping function of the laser motion required the pair of parallel devices to be placed at a 90-degree angle to one another as much as possible to decrease errors from this stepping motion. The orthogonal design as well as the placements of resistors such as the combined DLD waste resistor were placed below the DLD to counteract the settling of cells that would otherwise be trapped. In general, the use of a single DLD waste resistor rather than one for each parallel array was useful for saving space on the chip in addition to improving the performance of the DLD arrays. Another significant design change for the DLD arrays is that the device depth here was limited to 52 µm due to manufacturing limitations on wall angle. Essentially, devices with a significant wall angle cause excess geometric drift of particles diminishing DLD performance. As such, a ±2 µm tolerance on post-bond device height, and a ±1.5 deg tolerance on wall angle were identified as critical quality attributes for the devices. Excessive variation on device height would hinder IFD performance, while excessive variation in wall angle would significantly impact DLD cell separation. Specifications for one version of the chip (v1.3 M) including tolerances for different parameters are presented in Supplementary Table 5. The reduced depth diminished the throughput by 5 times compared to our previously published 150 µm deep silicon devices, and thus required the increased parallelization of the DLD array to 128 total arrays to preserve the high throughput.

The mastering and injection molding were carried out by Sony DADC Biosciences (Anif, Austria) using proprietary techniques. Briefly, Sony’s injection molding process is unique in that it involves a combination of injection, compression, and a highly-dynamic-variothermal approach, all under low-pressure conditions. Molten polymer is first injected into the mold as the mold is closing to enhance the filling capability. The mold is specially designed to have dedicated resistor elements that can very rapidly modify the local mold temperature distribution, thus optimizing flow of the molten polymer and enabling replication of high aspect-ratio features such as the DLD pillars.

Other significant design additions were necessary to improve the reliability of the devices. Most importantly, the filling of such a complex geometry and gas impermeable device is not trivial. A venting process is used that allows for minimal bubbles to be trapped within the chip during the priming process. Devices are currently primed with a 25% ethanol solution. Quantitative specifications around priming are still being developed. Large 40 µm filters initially external to the chip were later added on chip, which improved the reliability of the chip from 60 to 90% by removing cell/bead aggregates that would have clogged portions of the microfluidic channels. Also, the use of the cassette layer allows for the fluidics to be much easier to set up.

A brief overview of the history of the design is presented in Supplementary Figure 14. The most recent design is v1.4.4 is currently under testing and incorporates a 4.2x DLD based concentrator on the chip reducing the volume of the product significantly.

The approximated channel dimensions and associated flow speeds for the different channels are also available in the Supplementary Information.

### Sample Preparation

Blood samples were collected from patients and healthy volunteers following experimental protocols reviewed and approved by the Dana-Farber Cancer Institute IRB, in accordance with the applicable Federal guidelines and regulations. Dana–Farber/Harvard Cancer Center IRB-approved protocol (Protocol No: 05-300) was used to obtain informed consent from all patients and healthy donors. The expiration date for the protocol is 03/01/2018. Some healthy samples were also ordered from Research Blood Components, LLC (Brighton, MA).

Whole blood is labeled before it is processed with the CTC-iChip. Leukocyte-specific biotinylated antibodies (anti-CD45, anti-CD66b, and anti-CD16) and 1 μm magnetic beads are added based on the total volume of the blood sample. 180 µL of an antibody cocktail, consisting of 0.083 biotin-anti-CD45 mg/mL, 0.0083 biotin-anti-CD66b mg/mL, and 0.0083 biotin-anti-CD66b mg/mL to 0.1 mg/mL, is added per mL blood. The blood sample is then gently mixed on a sample rocker/rotator (HulaMixer sample mixer, Life Technologies, catalog #15920D) for at least 20 minutes but no longer than 2 hours. Whole blood is then incubated with 1.2 mg of streptavidin coated 1 µm magnetic Dynabeads® per mL blood, and incubated for a minimum of 25 minutes on the rocker/rotator. Based upon both CYTOF and other investigations it was determined that CD16 was the best addition to our leukocyte depletion panel.

### Preparation of Running Buffer

1% Pluronic-F68 (Sigma, P/N P1300) in 1x PBS buffer (Cellgro, P/N 21-040-CV) is filtered using a 0.2 µm pore Nalgene sterilization system (Fisher, P/N 09-740-25 A) prior to use.

### Dynabeads Preparation

Streptavidin-coated magnetic beads are used to label cells tagged with biotinylated antibodies. Beads are washed with 0.01% TWEEN 20 in 1xPBS to remove the storage solution, then washed in 0.1% BSA in 1xPBS to reduce nonspecific binding. These unconjugated beads are stored in 0.1% BSA at a working concentration of 10 mg Dynabeads per mL.

### Depletion Antibodies Preparation and Handling

Aliquots of biotinylated CD45/CD66b/CD16 were prepared at a total concentration of 1 mg/mL. Each aliquot contains the following antibodies: biotinylated anti-human CD45 (Veridex, clone HI30, IgG1, 0.833 mg/mL), biotinylated anti-human CD66b (AbD Serotec, clone 80H3, IgG1, 0.083 mg/mL), biotinylated anti-human CD16 (BD Biosciences, clone 3G8, IgG1, 0.083 mg/mL).

### CTC Enumeration, Sizing and Expression Measurements

Following processing of whole blood samples for CTC isolation with the CTC-iChip, CTCs were enumerated and characterized using an Amnis imaging flow cytometer (ImageStream X Mark II). For a typical 10 mL blood sample processed on the CTC-iChip, the product volume is 5.5 mL. Since the Amnis flow cytometer requires an input volume of 50–200 µL, the CTC-iChip product was first concentrated using a microfluidic concentration chip, which was designed and built in-house^44^. The concentrator is used to concentrate the sample by 50x, reducing the volume to 100 µL. Staining reagents are added to the concentrated sample and processed through the Amnis imaging flow cytometer without additional sample preparation (More details in Supplementary Information).

Using the most uniform surface stain for each cell type (WBC-CD45/CD16, Prostate-Lung-Breast – EpCAM, Melanoma – CD146) a threshold was used to eliminate background signal from the image and determine the perimeter of the cell. The area within this perimeter was used to calculate the diameter of the cell assuming a circular area. The membrane expression was determined through calculation of the mean pixel intensity within a membrane mask. The membrane mask was created by subtracting a perimeter mask that is eroded by 7 pixels (0.5 µm per pixel) from a perimeter mask that is dilated by 3 pixels (see Supplementary Figure 15). The mean pixel intensity value within this membrane mask is the membrane expression value reported. Note that these values are scaled by the laser power intensity measured on each experimental day. The plating and staining protocol for the data presented in Fig. 3 is given in the Supplementary Information.

### Cell Culture

Details on cell culture materials and practices are available for each cell line in the Supplementary Information.

### Cell Spike Counting Procedures

For each spiked cell run the cell line suspension was centrifuged at 201 RCF for 3 minutes and resuspended in 1x PBS with 1% F68. A 50 µL aliquot of the suspension is taken and stained with DyeCycle Green (1:10000). These are counted in two hemocytometers and then the stock solution is diluted to ensure a spike volume of 100 µL to achieve the desired cell concentration. An 450 µL aliquot of the diluted suspension is taken and again stained with DyeCycle Green (1:10000) which is then counted across four Nageotte chambers which are averaged giving the final spiked cell concentration.

### Quantitative Size and Fluorescence Calibration

Quantum**™** R-PE MESF beads (Bangs Laboratories) were run through the imaging flow cytometer using the same analysis protocol including staining. The size of the beads is overestimated by about 16%. As shown in Supplementary Figure 8 there is also an intensity dependence of this overestimation where low intensity particles are found to be smaller.

This led to a calibration relationship between our analysis method and the standard MESF measurement using flow cytometry. This relationship is presented in Supplementary Figure 8. While only two cell lines overlap, there is also an approximate linear relationship between flow cytometric data and our data where one a.u. of the mean membrane expression utilized here is approximately equal to 520 MESF^30^.

### CTC Scoring Using Multispectral Imaging

Detection of CTCs in breast and prostate patient samples was performed using an automated fluorescence multispectral microscopy-scanning platform (Vectra 2.0, Perkin Elmer) that employed a unique 5-color immunofluorescence assay panel for each cancer type. All clinical samples were imaged at empirically derived exposure times developed with spike cell samples as well as healthy donors and patients. Circulating tumor cells were identified and scored according to specific criteria (Supplementary Information Table 4) for each fluorescent marker against cellular/staining parameters developed with model systems using cancer cell lines positive for disease specific signals as well as blood cells. The criteria were embedded in an automated algorithm used by the Vectra platform to automatically identify and classify candidate CTC targets. All candidate CTCs were manually scored by two blinded human reviewers against the established criteria and subsequently tabulated to generate counts according to signal presence.

### CTC Analysis Using Mass Cytometry

20 milliliters of whole blood from a patient with castration-resistant prostate cancer was processed using CTC-iChip. The product was incubated with Rh conjugated DNA intercalator for live cell discrimination, centrifuged at 400 g for 5 minutes and resuspended in 100 ul of 0.5% BSA in PBS buffer with metal-conjugated antibodies for surface antigens (1 ul each). Following 30 minutes of incubation at room temperature, sample was washed with 0.5% BSA in PBS buffer twice, fixed with 2% formaldehyde solution for 10 minutes. Membrane permeabilization was performed with 0.3% tween-20 solution for 30 minutes at room temperature followed by a 1 hour incubation with antibodies against epitopes inside the cells. Sample was washed three times with 0.5% BSA in PBS and resuspended in 1 mL mili-Q water for CyTOF analysis. Data analysis was performed using Cytobank.

## Electronic supplementary material

Supplemental Information
