# Supplementary material for: Monolithic Chip for High-throughput Blood Cell Depletion to Sort Rare Circulating Tumor Cells
Source: Sci Rep. 2017 Sep 7;7:10936. doi: 10.1038/s41598-017-11119-x (PMC5589885; doi:10.1038/s41598-017-11119-x)
Supplement: Supplementary file 1 — Supplemental Information [file 41598_2017_11119_MOESM1_ESM.doc]

Supplemental Information for:

**Monolithic Chip for High-throughput Blood Cell Depletion to Sort Rare Circulating Tumor Cells**

Fabio Fachin^1*^, Philipp Spuhler^1*^, Joseph M. Martel-Foley^1*^, Jon F. Edd^1^, Thomas A. Barber^1^, John Walsh^1^, Murat Karabacak^1^, Vincent Pai^1^, Melissa Yu^1^, Kyle Smith^1^, Henry Hwang^1^, Jennifer Yang^1^, Sahil Shah^1^, Ruby Yarmush^1^, Lecia Sequist^2^, Shannon L. Stott^1,2^, Shyamala Maheswaran^1,2^, Daniel A. Haber^1,2^, Ravi Kapur^1^ and Mehmet Toner^1,3†^

*^1^BioMEMS Resource Center, Center for Engineering in Medicine and Surgical Services, Massachusetts General Hospital and Harvard Medical School, Boston, Massachusetts, 02114, USA*

*^2^Cancer Center, Massachusetts General Hospital, Boston, Massachusetts, 02114, USA*

*^3^Shriners Hospital for Children, Boston, Massachusetts, 02114, USA*

**These authors contributed equally to this work.*

^†^*Correspondence and requests for materials should be addressed to M.T. (email:* [*mehmet_toner@hms.harvard.edu*](mailto:mehmet_toner@hms.harvard.edu)*).*

**
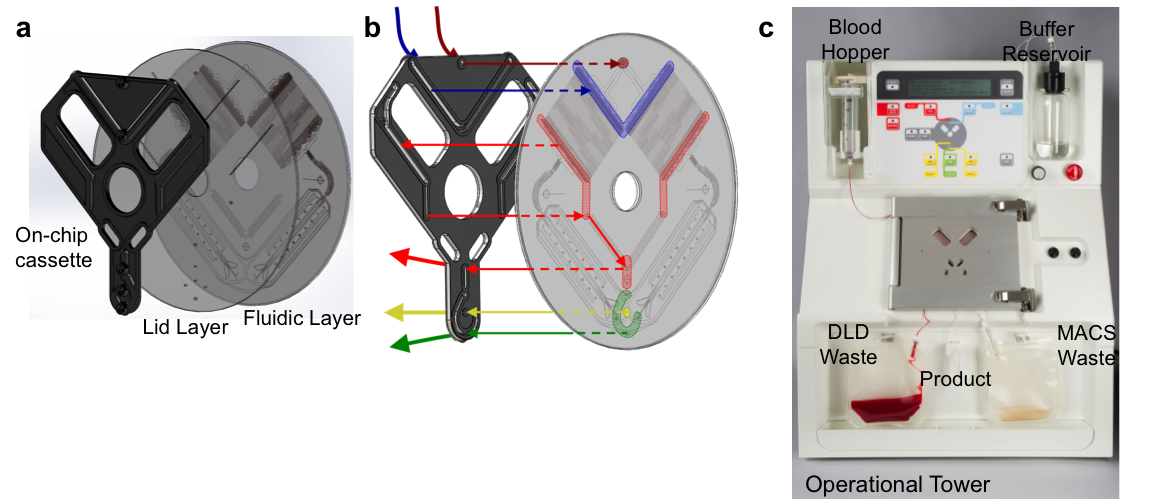
**

SI Figure 1: (a) 3D exploded view rendering of the isolator chip showing the three layers. (b) Labeled flow paths and how fluid moves between the layers. (c) Labeled image of the automated processing apparatus.

DLD Design Adjustments: Small adjustments were made to the design of the DLD arrays as a means of correcting for an asymmetry in the previous design caused by offset resets. Essentially, there was an imbalanced flow (phi) unaccounted for in between resets which was fixed.


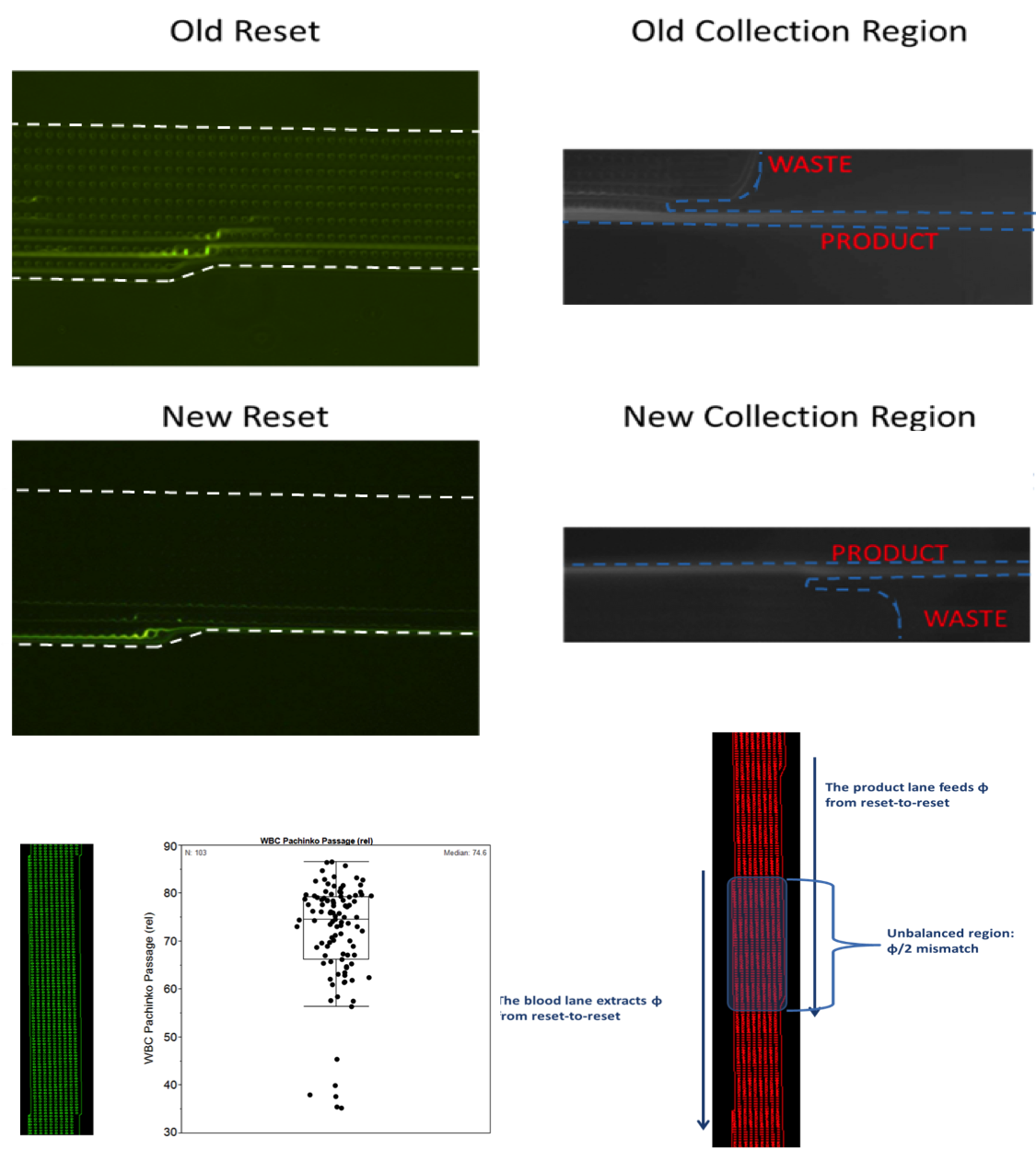


SI Figure 2: (a) Fluorescent streak images of calcein labeled white blood cells passing through the old (top) and now corrected (bottom) resets. (b) White blood cell passage statistics for new corrected design.


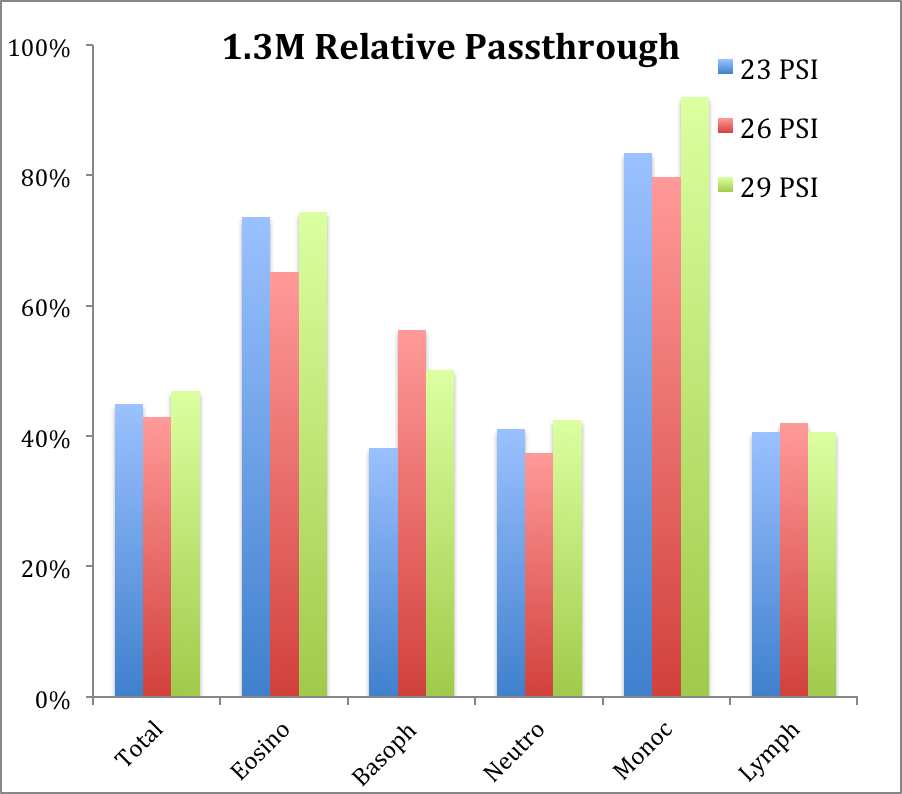


SI Figure 3: Percentage of different white blood cells passed through the DLD to the inertial focusing stages of the version 1.3M chip. This is data from a single patient with lower than average relative pass through of WBCs. The blood cell populations were measured using a standard automated complete blood count (CBC) system from Sysmex.

**DLD waste resistor design:**

The DLD product collection ratio was set to ensure collection of two fluidic “lanes” into the product streams. This is a result of the geometrical layout of the DLD array, where the product lane (lane “N”) and lane “N-1” comprise 22% of the total open cross sectional area at the DLD collection region. Collection of two lanes, rather than one, optimizes DLD yield by mitigating reset effects that could not be entirely mitigated. The effect of differential viscosity/HCT across the DLD lanes was accounted for in these flow rate calculations, with a range for sample HCT between 30-50% being acceptable for normal device operation.

Based on the 22% DLD target collection product ratio, the DLD waste resistor was designed to provide 22% of the total “downstream of DLD” resistance. As such, the DLD waste resistor was designed to provide a fluidic resistance 3.54 times lower than the IFD circuits, thus resulting in a final 22% product collection in the DLD circuit. The DLD waste resistor takes into account DLD multiplexing on each side of the disk.

The actual resistance of the DLD waste resistor was determined based on a combination of analytical approximations, finite element models and empirical observations. Due to the absence of an analytical model for wiggler-like features, the IF1 and IF2 resistances (as in Figure 1) were simulated in ANSYS using test structures whose pressure drops were then confirmed via experimental observation of relative volumetric outputs from the DLD waste resistor and IF stages. The resistances for MACS 1, MACS 2, and all other IFD waste, product, and connecting channels were analytically calculated using a 20-term series solution using Boussinesq’s published hydraulic resistance formulas and the correction factors associated with rectangular cross section channels [1].

**Manufacturing and Quality Controls:**

Correct chip operation requires that critical-to-quality (CTQ) parameters are maintained within nominal range during the entire sample processing. The following are some of the CTQs for the chip architecture presented herein: DLD sample injection ratio, DLD product collection ratio, IF flow rate, IF waste 1 ratio, IF waste 2 ratio, magnetic alignment, DLD pillar geometry, on-chip through-hole definition, and absence of large-area de-bonding. Where the first five CTQs in the above list are “fluidic-CTQs”, i.e. quantifiable fluidic parameters that can be monitored during sample processing and for which a direct correlation with device performance can be established (e.g., suboptimal cell focusing at excessively low IF flow rates), the latter four CTQs are “manufacturing-CTQs” that aim at quantifying and minimizing variability across different steps in the manufacturing process. For example, although bonding quality is a key factor to enable proper system operation, it is difficult to quantify the extent of de-bonding that will result in off-spec performance. As such, it is rather difficult to develop clear guard-bands around manufacturing CTQs, requiring extensive experimental campaigns to identify allowable limits. Conversely, fluidic CTQs are quantifiable and are directly related to device design, thus enabling the use of mathematical models to study their band-guards, as well as their dependence on, e.g., device geometrical properties such as channel depth. A lumped resistor model of the fluidic system herein is presented in SI Figure 14a, where each model resistor represents the parallel of several single components in the actual device (e.g., the “64 DLD” resistor represents the parallel of the 64 single DLD arrays on each disk half). The lumped model enables one to analyze and visualize the interdependencies between system subcomponents (see Jacobian matrix analysis in SI Figure 14b), as well as to analyze the sensitivity of each CTQs to selected parameters. As an example, in SI Figure 15 we plot the sensitivity of the five fluidic CTQs against a uniform disk-wide variation in channel depth (where 52µm is the nominal device depth), assuming a nominal operating pressure of 21psi (pressure at which the IF flow rate should equal 130µl/min). The numerical model predicts that all CTQs will remain within their allowable ranges (as indicated by the dashed red-lines) as long as the device depth remains within ~6µm (i.e., ~10% of the nominal 52µm specification), at which point the IF flow rate will fall outside its allowable range (100-160 µL/min).


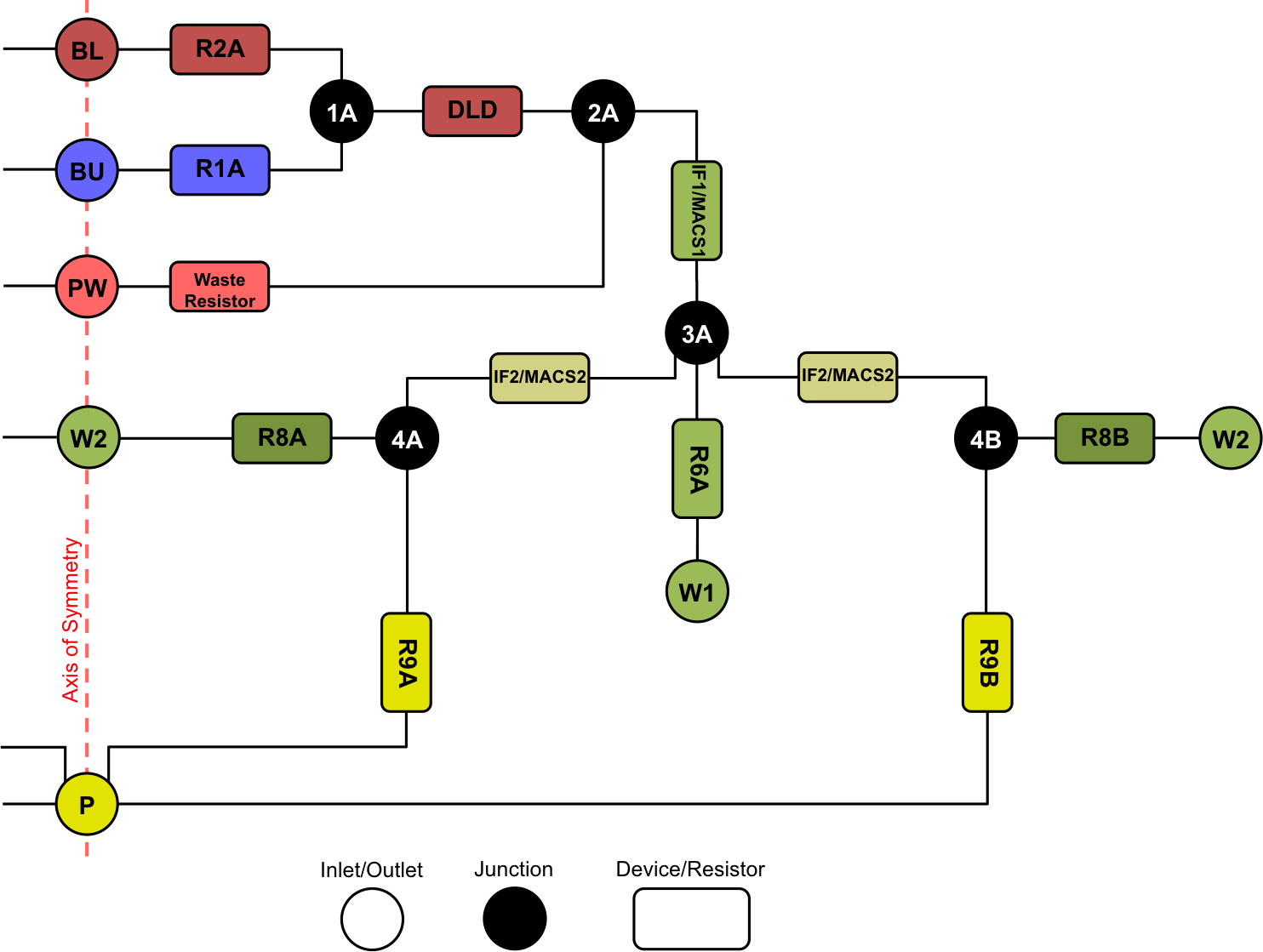

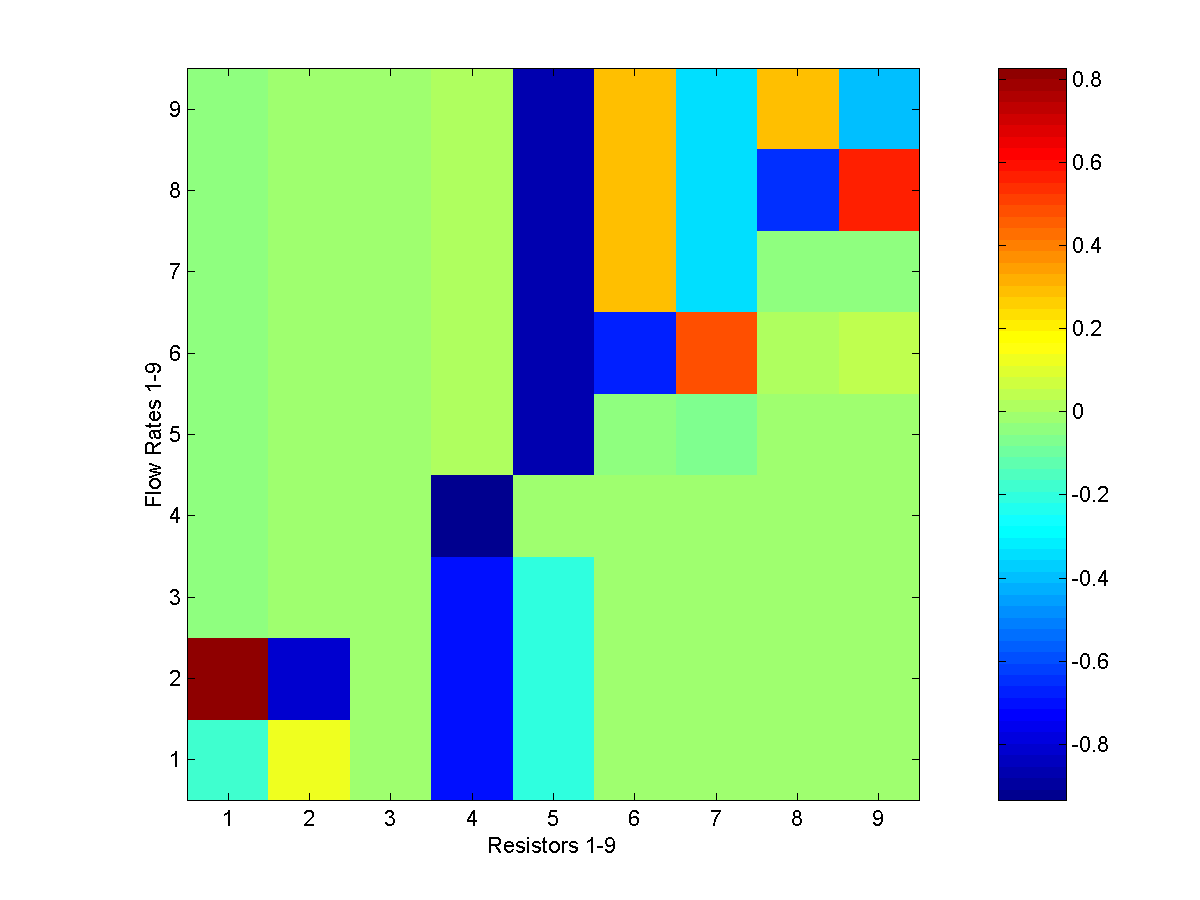


SI Figure 4: Resistor network (left) and resulting Jacobian matrix (right). 1: Pachinko buffer inlet, 2: Pachinko blood inlet, 3: Pachinko array, 4: Pachinko waste, 5: NIFD stage 1 wigglers, 6: NIFD stage 1 waste, 7: NIFD stage 2 wigglers, 8: NIFD stage 2 waste, 9: NIFD product. Positive values indicate a higher than nominal flow rate and negative values lower than nominal.


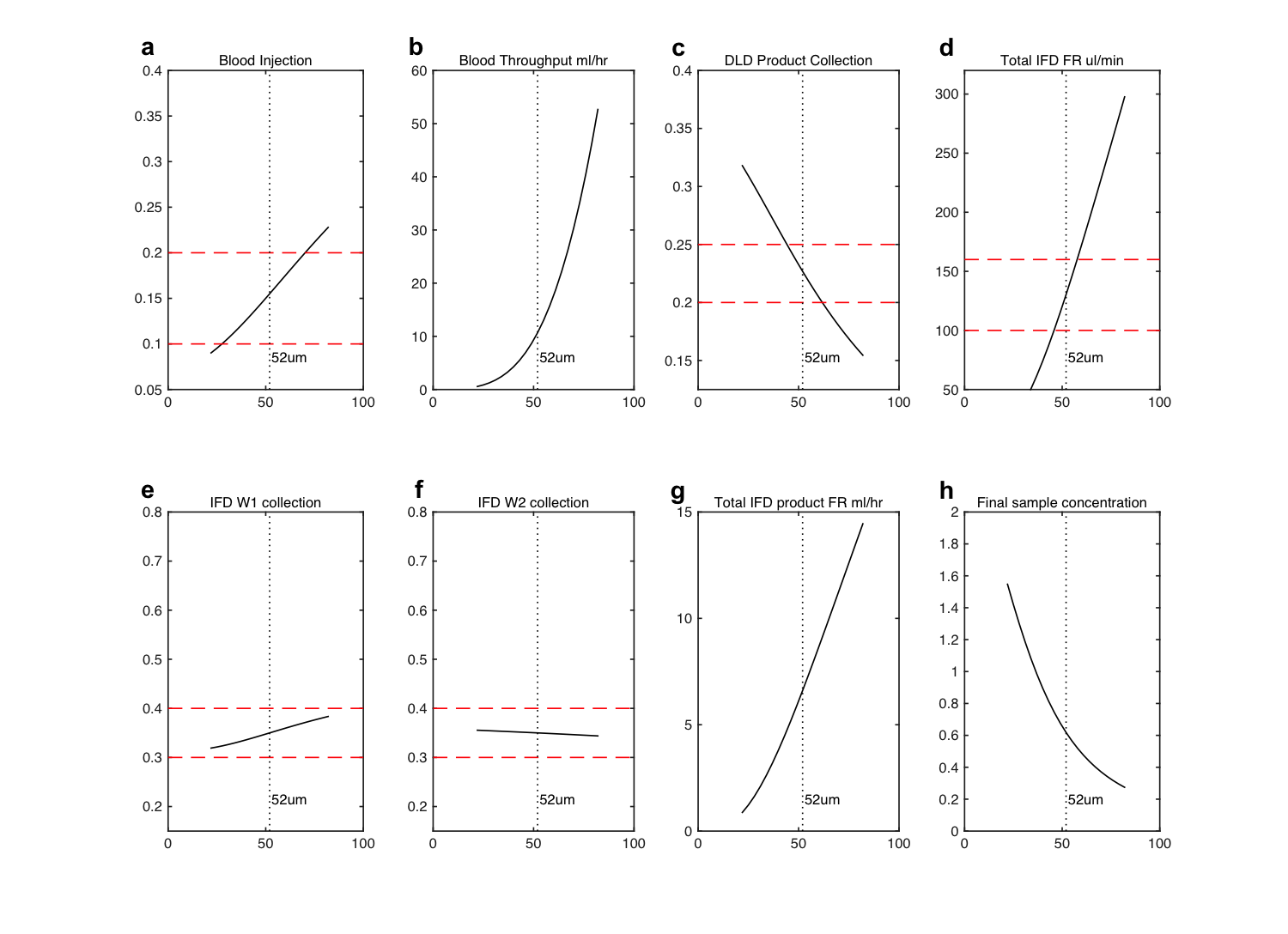


SI Figure 5: Critical to Quality (CTQ) analysis of the 5 fluidic based CTQs and the resulting overall flow rates investigating the uniform error in channel depth across the entire chip. (a) Blood injection ratio into DLD arrays, (b) The overall blood flow rate, (c) The DLD array collection ratio after separation, (d) the total flow rate into IF1, (e) the 1^st^ and (f) 2^nd^ IF waste collection ratios, (g) total monolithic iChip IF flow rate in mL/hr and (h) the dilution ratio of the final sample as compared to the input.

Depth uniformity:

All monolithic versions are designed for a depth of 52±2µm. Device depth must be within this range to ensure proper device operation; failure to do so will result in erroneous fluid distribution within the device, with possible risk of RBC carryover, low depletion, poor focusing and loss of target cells.

Bonding quality:

Bonding quality affects device performance by altering the fluid distribution within the device. Bonding must guarantee a perfect seal at all device locations, and it must not alter the local channel geometry. In this light, both over bonded and under bonded solutions are sub-optimal, as they may results in a local decrease / increase of channel cross section. Laminated solutions need to be assessed to guarantee that no significant bowing occurs under pressure operation. In general, bonding solutions need to be compatible with up to 40psi pressure conditions. More quantitative specifications around bonding need to be developed.

Pachinko array specifications:

The critical specifications for the pachinko arrays, including gap, reset gap replication, shift, array depth, and pillar-geometry were included in each Section outlining the specification for the different monolithic chip version. Feature wall angle should be maintained ≤1deg. All these specifications must be simultaneously satisfied.

Additionally, requirements on the acceptable array defect density (e.g., missing pillars, debris, protrusions) need to be defined for the Pachinko regions. The acceptable array defect density for silicon Pachinko chips has been defined and included in “Device Specification Document: MGH Chip “O212 Pachinko” Series - Manufacturing and Defect Specifications”. It is predicted that the specifications for the monolithic chip will not be more demanding than those for silicon devices.

Focus Quality:

Inertial focusing of rare cells is critical to the capture yield of CTCs. A shift in the focus position or the focus quality, defined by the full-width-half-max (FWHM) of the cell distribution along the cross section of the flow channel, results in a reduction in the rare cell capture yield. A safety margin of +/- 10% for the focus position and focus quality is incorporated into the design. Focus quality is primarily affected by RBC carryover to the IFD from the DLD. Figure 1 shows the streak image quality as a function of the RBC carryover. With 2-3 log depletion of RBCs (0.4 – 4% HCT), the streak quality remains acceptable, resulting in >95% capture of CTCs.

Antibody and bead quality and targeting sensitivity CTQ for 99.99% (4 log) leukocyte depletion:

The primary CTQs related to leukocyte depletion are reagent based labeling of leukocytes with a magnetic load required for deflection and the magnetic deflection of cells, which carry this minimum magnetic load, to the waste. In other words, the efficiency of leukocyte labeling in whole blood must be such that the fraction of leukocytes labeled with >=2 MyOne Dynabeads is greater than 99.99%.

Specificity CTQ for >95% capture yield of target CTCs:

The non-specific binding (NSB) of MyOne beads to CTCs can result in magnetic deflection of target CTCs to the waste. NSB of MyOne beads can result either from NSB of biotinylated antibodies to the target CTCs or NSB of the bead itself to the target CTC. The total NSB must be low enough to ensure that < 5% of target CTCs have >=2 MyOne Dynabeads per CTC.

Magnetic interfacing:

It is critical that the device is properly aligned to the magnetic circuit. Magnetic misalignment results in low depletion and possible loss of target cells. Alignment needs to be guaranteed both on xy chip plane (±150µm) and along the z-plane (+-150µm).


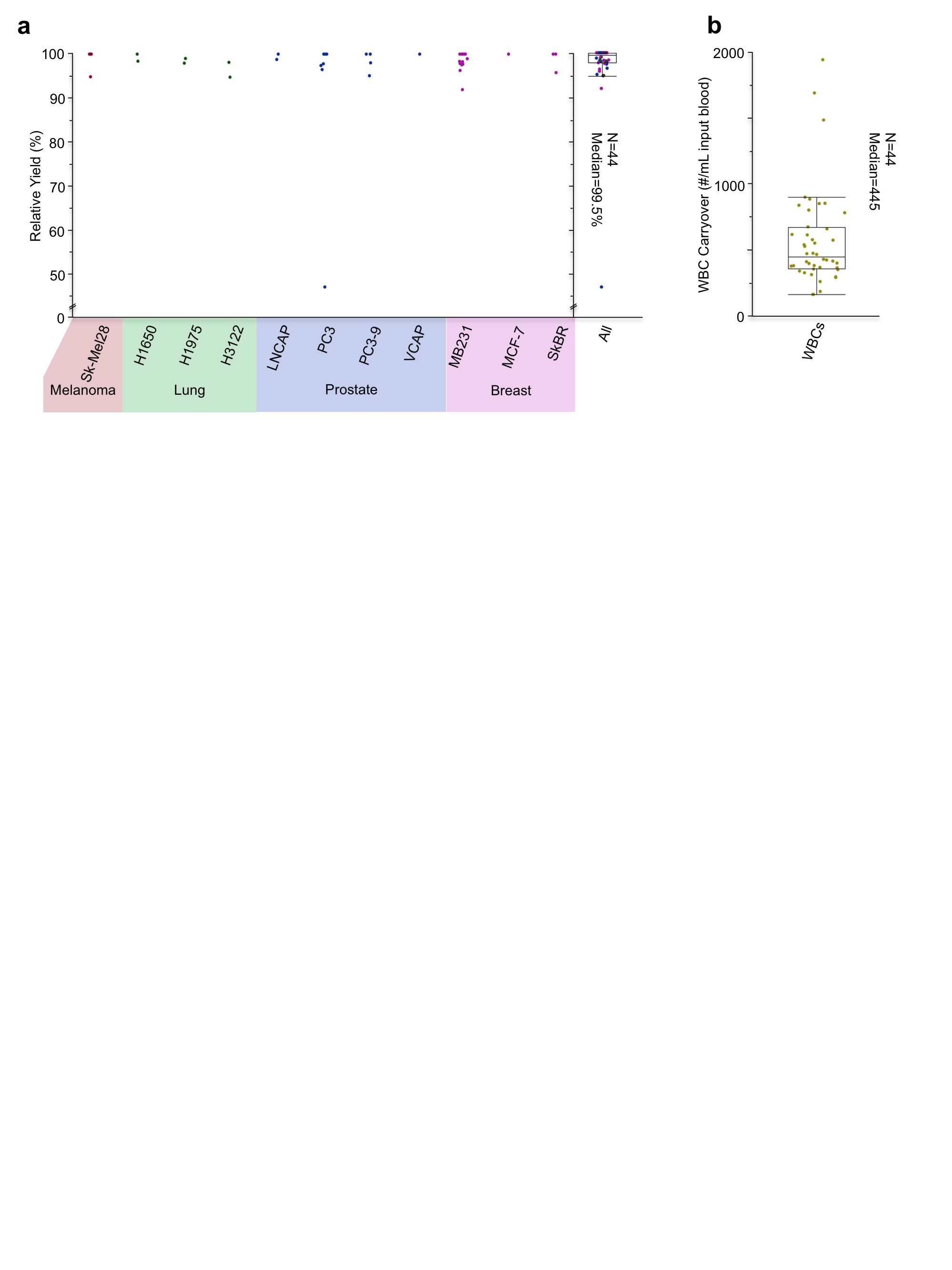


SI Figure 6: Yield and purity of spike cell lines in CTC-iChip across different cell lines. Sk-Mel28 (n=4), H1650 (n=2), H1975 (n=2), H1322 (n=2), LNCAP (n=2), PC3 (n=8), PC3-9 (n=4), VCAP (n=1), MB231 (n=15), MCF-7 (n=1) and SkBR (n=3).

SI Table 1: Spiked Sample Run Details

| Cell Line | Spiked Cell Concentration (Cells/mL) | Relative Yield (%) | Cell Line  Averages (%) | Disease  Average (%) |
| --- | --- | --- | --- | --- |
| SK-MEL-28 | 299 | 100.0 |  |  |
| SK-MEL-28 | 299 | 100.0 |  |  |
| SK-MEL-28 | 304 | 94.8 |  |  |
| SK-MEL-28 | 304 | 100.0 | 98.7 | 98.7 |
| H1650 | 19 | 100.0 |  |  |
| H1650 | 1960 | 98.4 | 99.2 |  |
| H1975 | 296 | 99.0 |  |  |
| H1975 | 289 | 97.9 | 98.5 |  |
| H3122 | 131 | 94.7 |  |  |
| H3122 | 116 | 98.1 | 96.4 | 98.3 |
| LNCAP | 20 | 100.0 |  |  |
| LNCAP | 577 | 98.8 | 99.4 |  |
| PC3 | 302 | 96.4 |  |  |
| PC3 | 302 | 100.0 |  |  |
| PC3 | 295 | 97.4 |  |  |
| PC3 | 591 | 100.0 |  |  |
| PC3 | 21 | 100.0 |  |  |
| PC3 | 22 | 46.3 |  |  |
| PC3 | 722 | 97.8 |  |  |
| PC3 | 722 | 100.0 | 92.2 |  |
| PC39 | 295 | 100.0 |  |  |
| PC39 | 295 | 100.0 |  |  |
| PC39 | 293 | 98.0 |  |  |
| PC39 | 222 | 95.0 | 98.3 |  |
| VCAP | 188 | 100.0 | 100.0 | 95.3 |
| MB231 | 349 | 100.0 |  |  |
| MB231 | 341 | 100.0 |  |  |
| MB231 | 341 | 100.0 |  |  |
| MB231 | 114 | 97.5 |  |  |
| MB231 | 40 | 100.0 |  |  |
| MB231 | 300 | 98.3 |  |  |
| MB231 | 150 | 98.9 |  |  |
| MB231 | 75 | 97.7 |  |  |
| MB231 | 294 | 98.3 |  |  |
| MB231 | 283 | 100.0 |  |  |
| MB231 | 283 | 100.0 |  |  |
| MB231 | 400 | 100.0 |  |  |
| MB231 | 448 | 91.8 |  |  |
| MB231 | 448 | 97.8 |  |  |
| MB231 | 284 | 96.2 | 98.4 |  |
| MCF-7 | 21 | 100.0 | 100.0 |  |
| SKBR | 330 | 100.0 |  |  |
| SKBR | 330 | 95.7 |  |  |
| SKBR | 5000 | 100.0 | 98.6 | 98.5 |

SI Table 2: Patient Details

| No. | Patient ID | Date of Sample Draw | Other Information | CTCs | CTCs/mL (equivalent blood volume) ** |
| --- | --- | --- | --- | --- | --- |
| 1 | Mel85 | 3/27/14 | MEL-85: Stage IV M1c, BRAF-mutant melanoma. | 25 | 1.5 |
| 2 | Mel86 | 3/25/14 | MEL-86: Stage IV M1c, NRAS-mutant melanoma. | 8 | 0.9 |
| 3 | TH134a | 2/4/14 |  | 63 | 63.0 |
| 4 | TH134b | 3/11/14 |  | 1 | 0.1 |
| 5 | TH174a | 1/9/14 |  | 2 | 0.7 |
| 6 | TH174b | 1/29/14 |  | 3 | 0.8 |
| 7 | TH192 | 2/13/14 |  | 6 | 1.9 |
| 8 | TH207 | 1/15/14 |  | 3 | 1.4 |
| 9 | TH217 | 1/16/14 |  | 1 | 0.4 |
| 10 | TH223 | 3/12/14 |  | 7 | 0.8 |
| 11 | TH78 | 3/5/14 |  | 6 | 1.6 |
| 12 | GU150 | 8/21/14 | Stage IV (metastatic) | 7 | N/A* |
| 13 | GU179 | 8/21/14 | Stage IV (metastatic) | 906 | N/A* |
| 14 | BR18 | 2/14/14 | IV | 41 | 9.6 |
| 15 | BR29b | 3/20/14 | IV | 12 | 1.2 |
| 16 | BR42 | 11/18/13 | IV | 172 | 20.9 |
| 17 | BR55 | 11/1/13 | IV | 3 | 0.2 |
| 18 | BR56 | 2/6/14 | IV | 65 | 15.9 |
| 19 | BR65 | 11/14/13 | IV | 12 | 1.4 |
| 20 | BR69 | 11/1/13 | IV | 428 | 62.1 |
| 21 | BR70 | 3/18/14 | IV | 2 | 0.3 |
| 22 | BR71 | 3/24/14 | IV | 5 | N/A* |
| 23 | BR72 | 11/21/13 | IV | 31 | 5.2 |
| 24 | BR82a | 11/25/13 | IV | 154 | 34.8 |
| 25 | BR82b | 12/16/13 | IV | 88 | N/A* |
| 26 | BR82c | 1/28/14 | IV | 1 | N/A* |
| 27 | BR89 | 12/4/13 | IV | 4 | 1.1 |
| 28 | BR93 | 10/31/13 | IV | 15 | 2.5 |
| 29 | BR98 | 2/10/14 | IV | 4 | 0.8 |
| 30 | BR99 | 11/5/14 | IV (pt. deceased 2/2014) | 38 | N/A* |
| 31 | BR104 | 12/10/13 | IV | 162 | N/A* |
| 32 | BR108 | 10/3/13 | IV | 3 | 0.6 |
| 33 | BR109 | 11/18/13 | IV | 179 | 19.2 |
| 34 | BR113 | 11/7/13 | IV | 35 | 4.8 |
| 35 | BR115 | 11/15/13 | IV | 23 | 3.2 |
| 36 | BR120 | 2/3/14 | IV | 11 | 4.0 |
| 37 | BR131 | 1/28/14 | III/IV "Locally advanced/metastatic" | 17 | 2.1 |
| 38 | BR134 | 2/26/14 | IV | 6 | 1.3 |

*Not Available

**Full sample was not always run through imaging flow cytometry analysis. The equivalent blood volume is in terms of the initial patient sample blood.


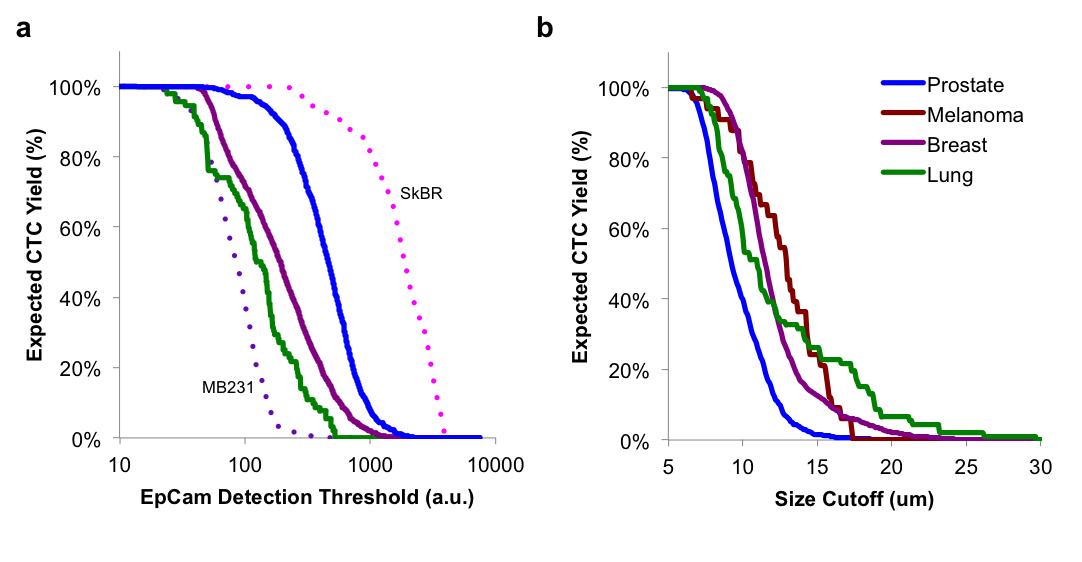
SI Figure 7: Waterfall plots indicating the expected CTC yield of (a) EpCAM and (b) size based technologies using the populations detected in this paper.

**Cell Size and Expression Quantification Details:**

Every detection system has biases and issues. Outlined here are a few limitations of imaging flow cytometry for measuring size. While brightfield or forward scatter is implemented for sizing in traditional flow cytometry, it is difficult to use in imaging flow cytometry especially given that many cells come through in pairs or overlapping in the same image. In order to only measure the CTCs, the fluorescence signal for EpCAM or CD146 was used for this measurement. As such we have to acknowledge that we may not be detecting extremely low expressing cells. This could mean that patient CTC numbers are actually even greater than portrayed. The focus quality of the images also can affect the size measured for the particle. So in general, the imaging technique presented overestimates the size of particles greater than ~100 a.u. expression by 16% and 35.6% of the particles below 100 a.u. which were detected in bright field were undetected by fluorescence. No pixel saturation was detected with the equivalent laser power settings as compared to cells. A single a.u. is equivalent to approximately 519 MESF (mean intensities were calculated not including beads undetected by fluorescence). The mean diameters for each type from 1 to 4 were found to be 1.05µm, 6.71µm, 8.86µm and 8.74µm, respectively (not including beads undetected by fluorescence). The true size of the calibration beads according to the manufacture was on average 7.65 µm.


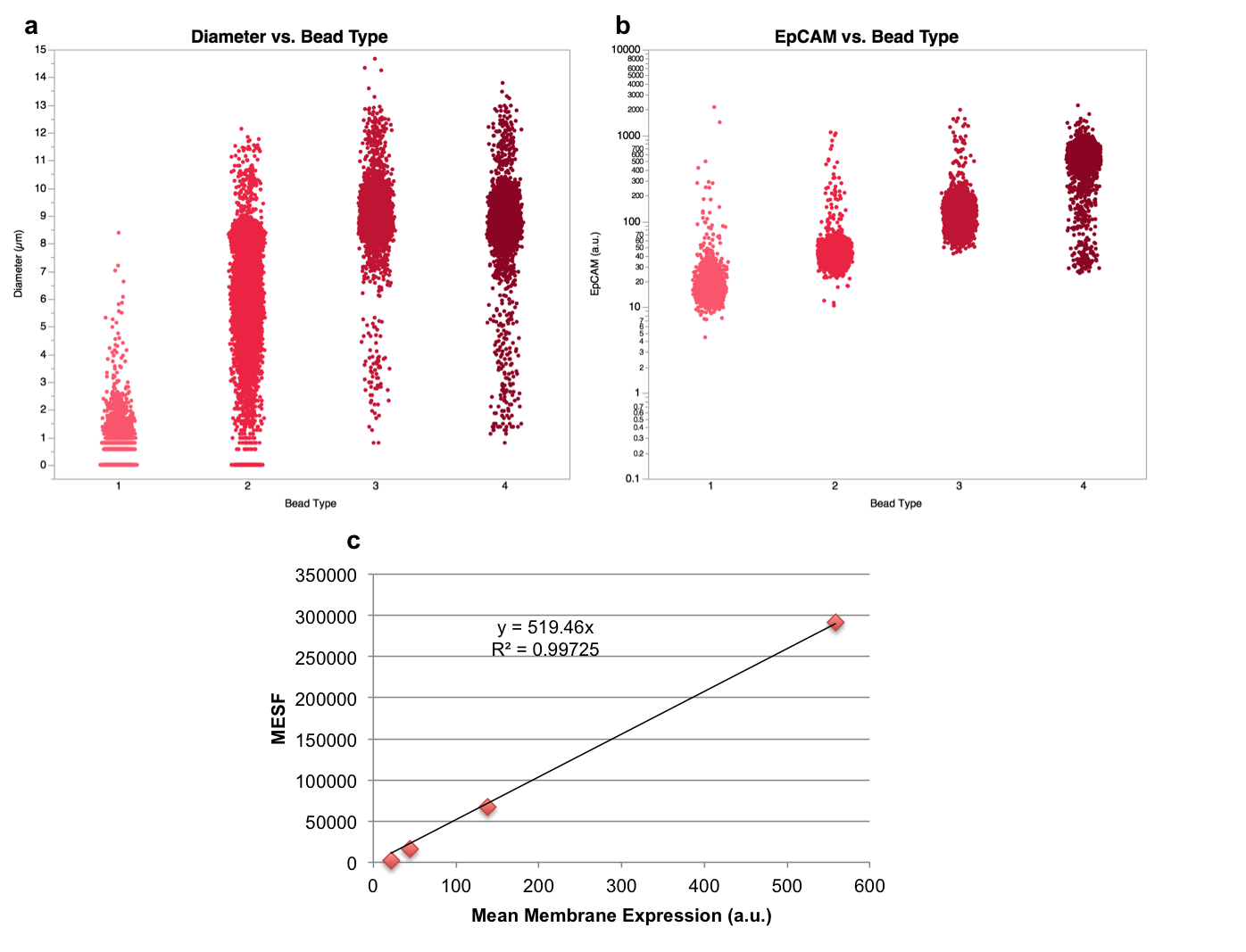


SI Figure 8: Accuracy of size and fluorescence quantification. (a) The relationship between the particle size and the intensity of the fluorescence calibration beads. Bead Type 1 - 1988 MESF, Type 2 - 16694 MESF, Type 3 - 67310, Type 4 - 291866. (b) The measured intensities of beads in equivalent units to cell measurements. (c) Population correlation between the means of measured fluorescence intensities and MESF values from manufacturer (Bangs Laboratories).

SI Table 3: Cell Search® results from literature.

| **Cell Type** | **Yield (Spike )** | **Reference** |
| --- | --- | --- |
| SKBr-3 | >/=85% (4 to 1142 cells into 7.5 mL) | Allard et al. CCR 2004. |
| PC-3 | 40% (20 to 300 into 7mL) | Harb et al. Trans Onc. 2013 |
| CAL-120 | 42% (500/mL) | Punnoose et al. Plos One. 2010. |
| SKBr-3 | >/=75% (500/mL) | Punnoose et al. Plos One. 2010. |
| MDA-MB-435 | 0% (50-150 into 7.5mL) | Sieuwerts et al. JNCI. 2009. |
| MDA-MB-436 | 0% (50-150 into 7.5mL) | Sieuwerts et al. JNCI. 2009. |
| SKBr-7 | 0% (50-150 into 7.5mL) | Sieuwerts et al. JNCI. 2009. |
| Hs578T | 0% (50-150 into 7.5mL) | Sieuwerts et al. JNCI. 2009. |
| BT549 | 0% (50-150 into 7.5mL) | Sieuwerts et al. JNCI. 2009. |
| MDA-MB231 | 12% (50-150 into 7.5mL) | Sieuwerts et al. JNCI. 2009. |
| MDA-MB-468 | 31% (50-150 into 7.5mL) | Sieuwerts et al. JNCI. 2009. |
| BT20 | 40% (50-150 into 7.5mL) | Sieuwerts et al. JNCI. 2009. |
| SUM149PT | 44% (50-150 into 7.5mL) | Sieuwerts et al. JNCI. 2009. |
| SUM149PE | 65% (50-150 into 7.5mL) | Sieuwerts et al. JNCI. 2009. |
| HCC1937 | 61% (50-150 into 7.5mL) | Sieuwerts et al. JNCI. 2009. |
| MPE600 | 53% (50-150 into 7.5mL) | Sieuwerts et al. JNCI. 2009. |
| CAMA-1 | 75% (50-150 into 7.5mL) | Sieuwerts et al. JNCI. 2009. |
| T47D | 75% (50-150 into 7.5mL) | Sieuwerts et al. JNCI. 2009. |
| LNCaP | ~60% (200 into 7.5mL) | Cann et al. Plos One. 2012 |
| SKBR-3 | 38% (100 to 10000 in 7.5mL) | Patel et al. Oncotarget. 2011 |
| SKBr-3 | 81% (4-12 cells in 7.5mL) | Riethdorf Clin Can Res. 2007 |


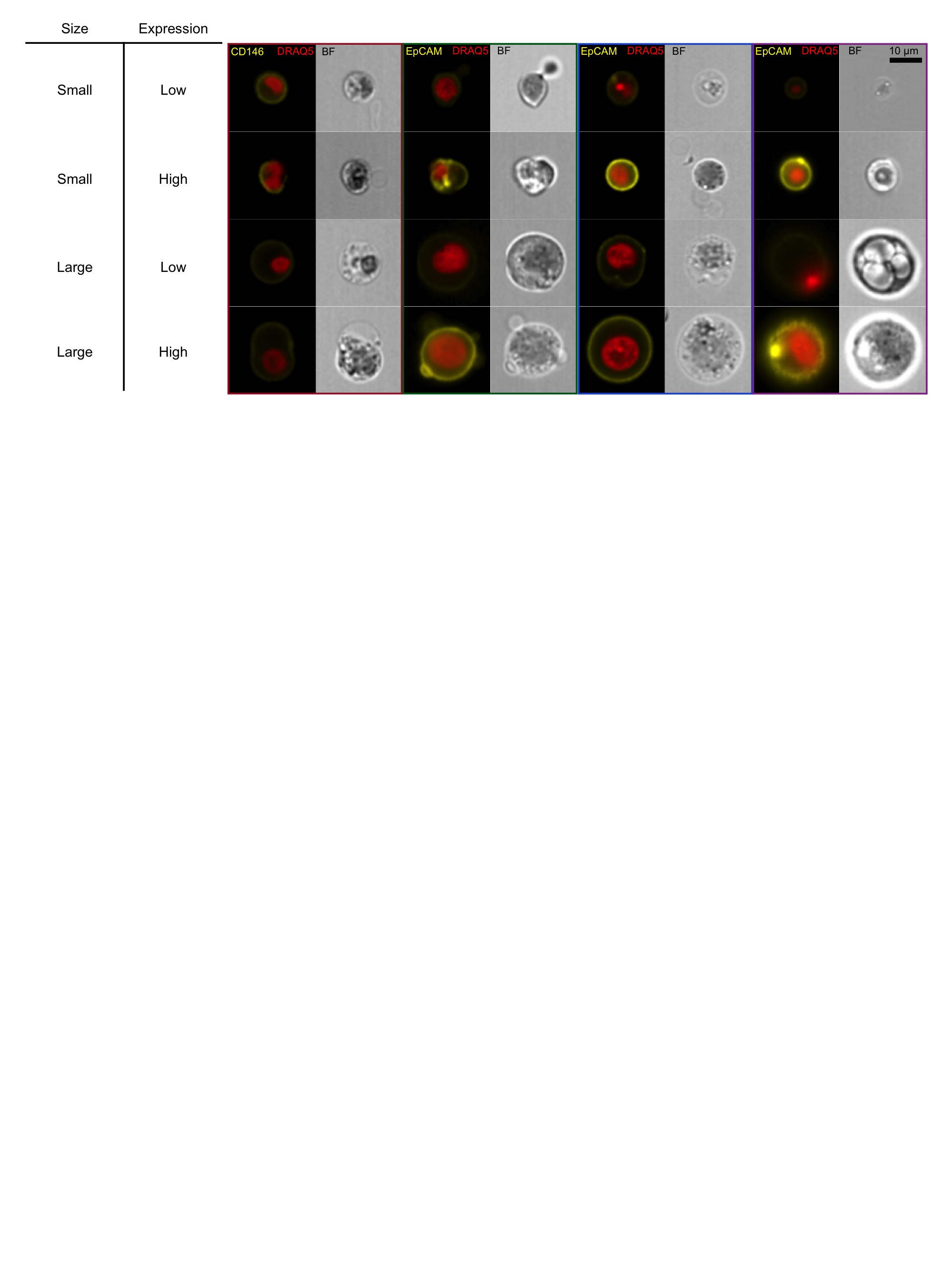


SI Figure 9: Example imaging flow cytometry images for patients highlighted in the main article Figure 2.

**Plated and Stained Imaging Analysis Using Multispectral Imaging:**

Detection of CTCs in breast and prostate patient samples was performed using an automated fluorescence multispectral microscopy-scanning platform (Vectra 2.0, Perkin Elmer) that employed a unique 5-color immunofluorescence assay panel for each cancer type. All clinical samples were imaged at empirically derived exposure times developed with spike cell samples as well as healthy donors and patients. Circulating tumor cells were identified and scored according to specific criteria (SI Table 4) for each fluorescent marker against cellular/staining parameters developed with model systems using cancer cell lines positive for disease specific signals as well as blood cells. The criteria were embedded in an automated algorithm used by the Vectra platform to automatically identify and classify candidate CTC targets. All candidate CTCs were manually scored by two blinded human reviewers against the established criteria and subsequently tabulated to generate counts according to signal presence.

SI Table 4: Criteria for scoring circulating tumor cells.

The absence of other stains in DAPI+ only events indicates that cells into fall into three different categories. These events can either be free nuclei ejected from WBCs, CTCs or nucleated RBCs or intact WBCs that have weak or absent expression of CD45, CD16 or CD66b surface antigens. Additionally, the cells can also be intact CTCs that may also have weak or absent antigen expression typical of CTCs undergoing epithelial to mesenchymal transition (EMT). Finally, the cells could also be CTCs cloaked with activated platelets whose close proximity to the cell could the diffusion and labeling of fluorophore labeled antibodies. The negative depletion scheme embodied in the iChip has the potential to enrich for all of the above, however we have found most of the DAPI+ only events are intact WBCs that have weak or absent expression of surface antigens as evidenced with advanced imaging flow cytometry that incorporates forward and side scatter detection which provide general phenotyping capabilities for classifying WBCs.


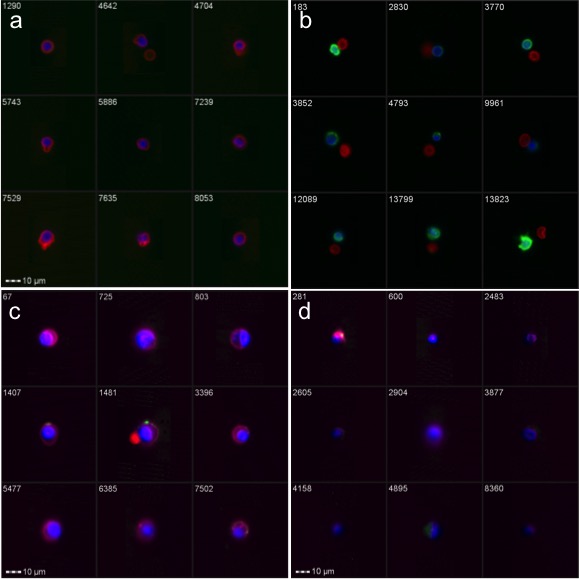


SI Figure 10: Example images of different nucleated event types found in product from healthy donor runs and analyzed using the deconvolution assay. Remaining leukocytes (DRAQ5+ / CD45+ / CD16+ / Glycophorin A- / Concanavalin A- / CD41-) not pictured, (a) erythroblasts (DRAQ5+ / Glycophorin A + / CD45- / CD16- / Concanavalin A- / CD41-), (b) bare nuclei (DRAQ5+ / Concanavalin A+ / Glycophorin A - / CD45- / CD16- / CD41-), and (c) megakaryocytes (DRAQ5+ / CD41+ / Concanavalin A- / Glycophorin A- / CD45- / CD16-) as well as (d) unclassified items.


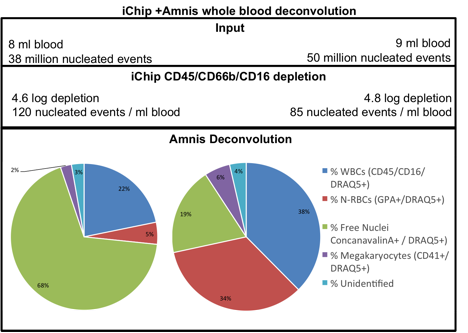


SI Figure 11: Nucleated events remaining in isolator product analyzed using a deconvolution assay. For these two healthy donors, >90% of the contaminant nucleated events in the product were classified with the assay and a majority of the nucleated events were identified as either free nuclei or nucleated RBCs.

The purity of the product in this system is one area for improvement as it is still below what is necessary for cutting edge sequencing technologies. However, as stated earlier, the targets necessary are already mapped out with the majority of contamination coming from bare nuclei, nucleated RBCs and megakaryocytes. Hopefully, the future targeting of these populations improves the purity as significantly as the addition of CD16, for targeting granulocytes, to the current assay. This is important for depletion of WBCs from cancer patient blood samples, since the granulocyte counts are heightened in patient blood relative to healthy donor blood samples. Even after depleting millions of background WBCs, the ~500-1000 nucleated events per mL remaining are such a challenge to remove. The possibilities are endless if the product from these samples can be analyzed directly through genetic sequencing, as this enables rapid personalized therapies. It is also clear that we may be losing some smaller and more deformable cells in the DLD arrays considering the WBC yield is only approximately 70%. While an increased pass through of nucleated cells may yield more CTCs it will also likely increase the carryover of contaminating WBCs. A greater understanding of the effects of deformability and how to account for such properties in the design of these arrays could improve the performance.

**Mass Cytometry Measurements of Remaining White Blood Cell Populations from previous version of iChip:**

40 milliliters of whole blood from a healthy donor was processed using isolator (only CD45 and CD66b antibodies used for depletion, a control whole blood aliquot run without antibodies was also processed). Sample was incubated with Rh conjugated DNA intercalator for live cell discrimination, centrifuged at 400g for 5 minutes and resuspended in 100 µl of 0.5% BSA in PBS buffer with metal-conjugated antibodies (1µl each). Following 30 minutes of incubation at room temperature, sample was washed with 0.5% BSA in PBS buffer twice, fixed with 2% formaldehyde solution for 10 minutes. Membrane permeabilization and DNA labeling were performed with 0.3% saponin solution and Ir conjugated DNA intercalator. Sample was washed three times with 0.5% BSA in PBS and resuspended in 1mL mili-Q water for CyTOF analysis. Data analysis was performed using Cytobank.


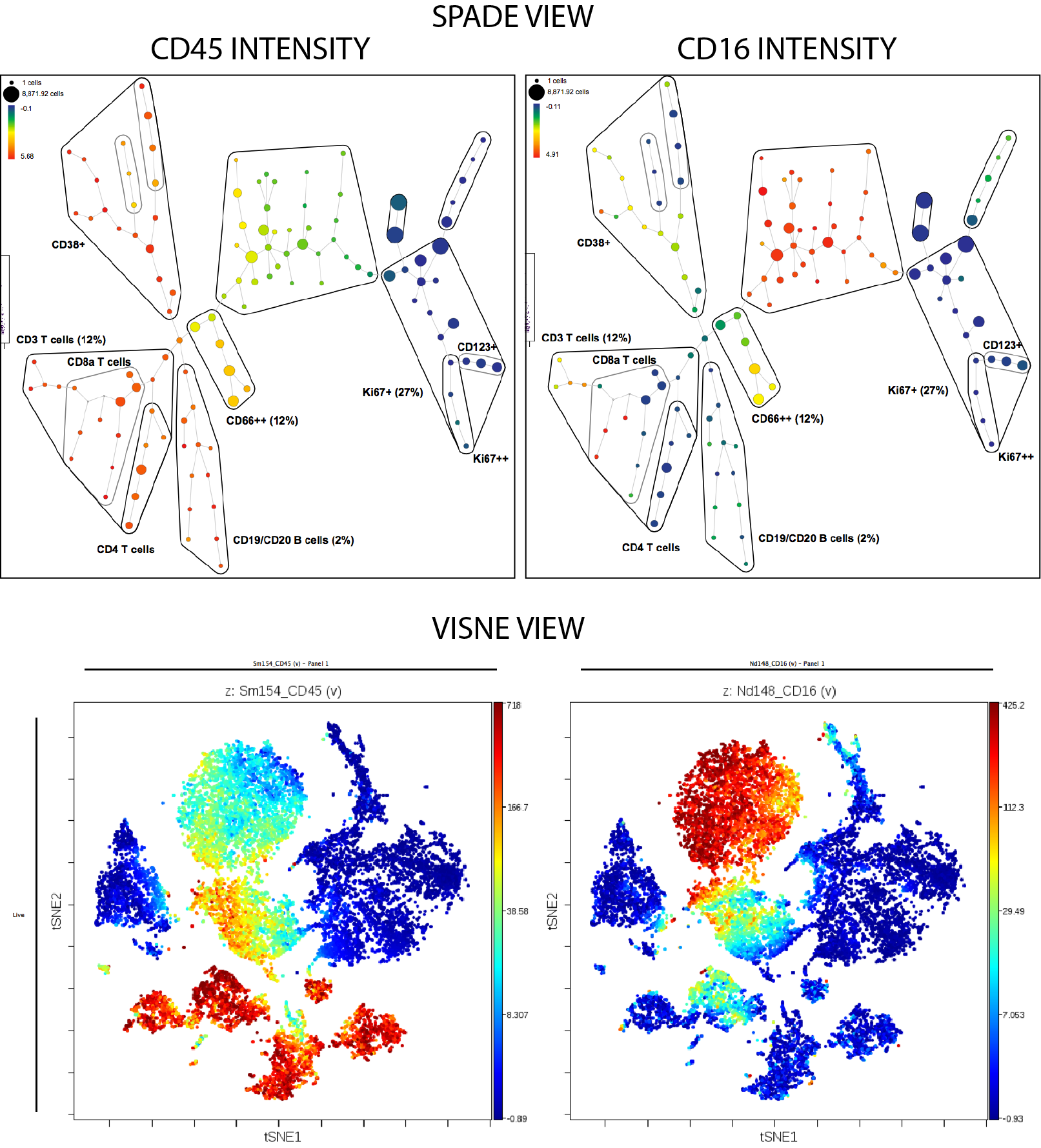


SI Figure 12: SPADE and viNSE views of two select markers from the 17 marker panel for identification of remaining leukocytes in isolator product. Visne clustering (viSNE enables visualization of high dimensional single-cell data and reveals phenotypic heterogeneity of leukemia. Amir el-AD et al. Nat Biotechnol (2013) 31(6):545-52) was performed using Cytobank for visualization of the leukocytes classified according to their markers.

SI Figure 13: viSNE views of 17 markers for isolator product used for investigating depletion cocktail improvement (viSNE enables visualization of high dimensional single-cell data and reveals phenotypic heterogeneity of leukemia. Amir el-AD et al. Nat Biotechnol (2013) 31(6):545-52) was performed using Cytobank for visualization of the leukocytes classified according to their markers.

SI Table 5: Selected specifications and tolerances for v1.3M chip


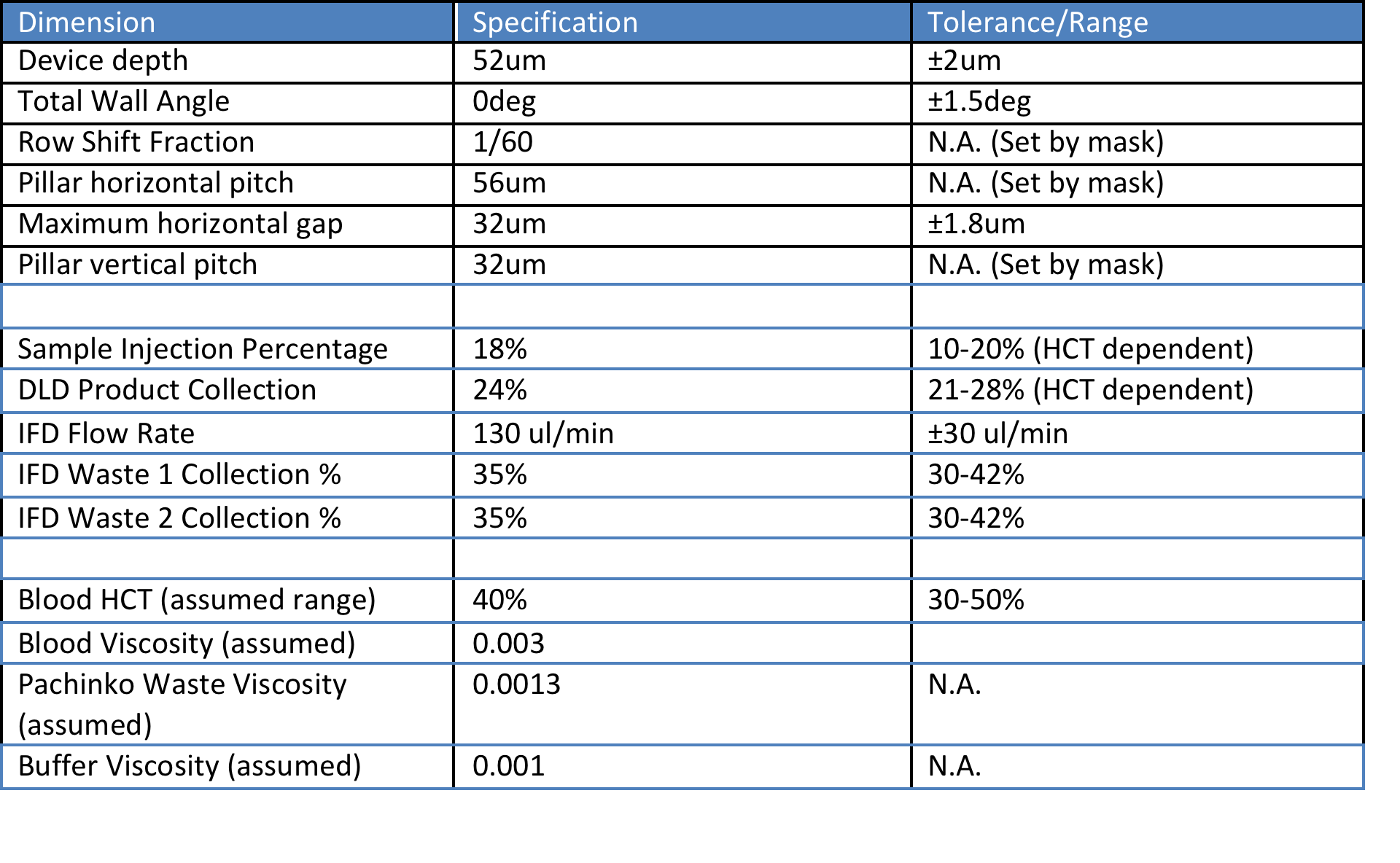


**IFD related specs and tolerance:**

Focus position is flow rate cell size dependent. The tolerance for flow rate and waste 1 / waste 2 collection was set based on empirical measurement of focus position for the range of flow rate and range of cell size, in order to ensure >95% capture efficiency for CTCs with size from 8 um to 50 um.


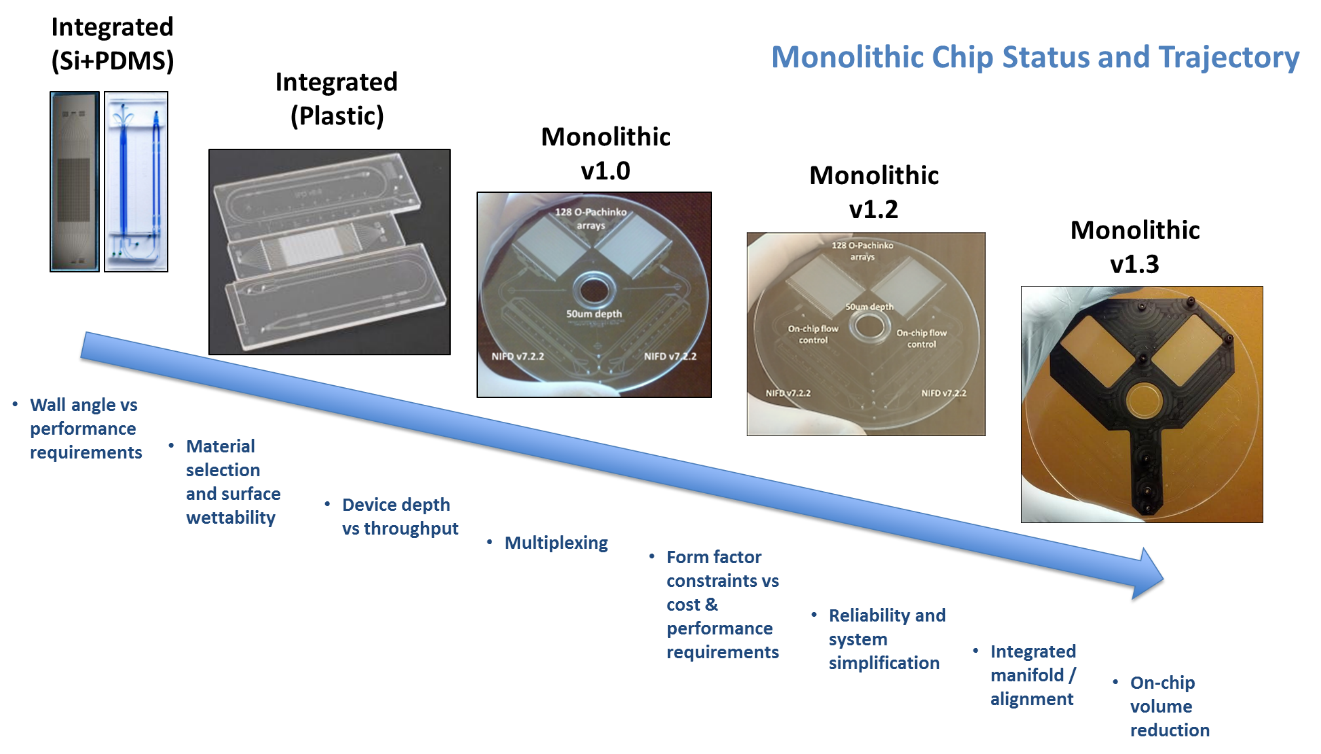


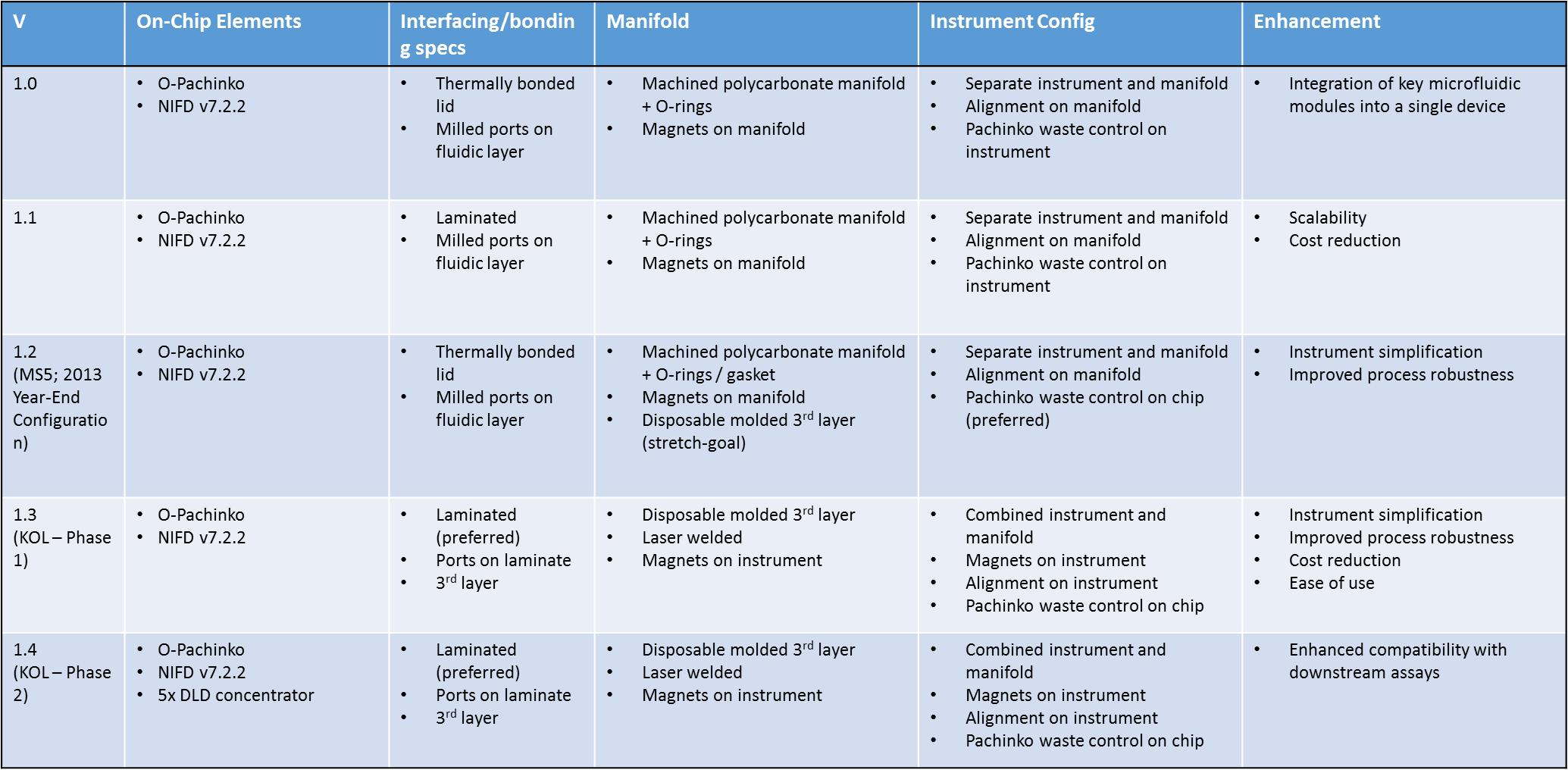


SI Figure 14: Chip Design History

SI Table 6: Approximate operational and geometric properties for different subsections of the devices used. The total estimated residence time in the monolithic-iChip is 7.4 seconds. The total estimated residence time in the microfluidic concentrator is 0.3 seconds.

|  | DLD | Converging | IF1 | MACS1 | IF2 | MACS2 | To Product Port | | Concentrator |
| --- | --- | --- | --- | --- | --- | --- | --- | --- | --- |
| Flow Rate (uL/min) | 31 | 130 | 130 | 260 | 85 | 85 | 55 | 55 | 500 |
| Channel Height (um) | 52 | 52 | 52 | 52 | 52 | 52 | 52 | 52 | 52 |
| Channel Width (um) | 2000 | 1000 | 75 | 1000 | 75 | 500 | 500 | 1000 | 750 |
| Channel Length (mm) | 20 | 10 | 25 | 37 | 5 | 37 | 40 | 15 | 65 |
| Cross Sectional Area (mm^2) | 0.10 | 0.05 | 0.00 | 0.05 | 0.00 | 0.03 | 0.03 | 0.05 | 0.04 |
| Average Flow Speed (mm/s) | 5.01 | 41.67 | 555.56 | 83.33 | 361.11 | 54.17 | 35.21 | 17.60 | 213.68 |
| Residence Time (s) | 3.99 | 0.24 | 0.05 | 0.44 | 0.01 | 0.68 | 1.14 | 0.85 | 0.30 |

**Amnis Staining Protocol:**

All stains are added 30 minutes prior to sample being run through Amnis ImageStreamX except DRAQ5 which is added 15 minutes prior. Assay #1 is used for all prostate and lung patients and associated cell lines. Assay #2 is used for all breast patients and associated cell lines. Assay #3 is used for all melanoma patients and Assay #4 for associated cell lines. Assay #5 is used to determine the relative populations of nucleated events that carry over into the product from a healthy donor sample. The volumes are added to final sample volume of 200µL and scaled accordingly for smaller samples.

SI Table 7: Stain details for Amnis Imaging Flow Cytometry

| **Assay #1 – Prostate and Lung** | | | | | |
| --- | --- | --- | --- | --- | --- |
| **Excitation laser power: 405 nm: 80 mW | 488 nm: 100 mW | 642 nm: OFF | 785 nm: OFF** | | | | | |
| **Channel** | **Antibody** | **Fluor** | **Vendor** | **Volume** | **Part Number** |
| Ch01 | CD45/16 | Pacific Blue | Invitrogen | 1.5 / 1 µL | MHCD4528/ MHCD1628 |
| Ch02 | EGFR | FITC | Veridex | 2 µL |  |
| Ch03 | EpCAM | PE | Invitrogen | 2 µL | A15782 |
| Ch04 | BF | | | | |
| Ch05 | DRAQ5 | | BioStatus | 3.5 µL (of 1:25) | DR50200 |
| Ch06 | (not used) |  |  |  |  |
|  |  |  |  |  |  |
| **Assay#2 - Breast** | | | | | |
| **405 nm: 80 mW | 488 nm: 100 mW | 642 nm: OFF | 785 nm: OFF** | | | | | |
| **Channel** | **Antibody** | **Fluor** | **Vendor** | **Volume** | **Part Number** |
| Ch01 | CD45/16 | Pacific Blue | Invitrogen | 1.5 / 1 µL | MHCD4528/ MHCD1628 |
| Ch02 | HER2 | FITC | BioLegend | 2 µL | 324404 |
| Ch03 | EpCAM | PE | Invitrogen | 2 µL | A15782 |
| Ch04 | BF | | | | |
| Ch05 | DRAQ5 | | BioStatus | 3.5 µL (of 1:25) | DR50200 |
| Ch06 | (not used) |  |  |  |  |
|  |  |  |  |  |  |
| **Assay#3 - Melanoma** | | | | | |
| **405 nm: 80 mW | 488 nm: 100 mW | 642 nm: OFF | 785 nm: OFF** | | | | | |
| **Channel** | **Antibody** | **Fluor** | **Vendor** | **Volume** | **Part Number** |
| Ch01 | CD45/16 | Pacific Blue | Invitrogen | 1.5 / 1 µL | MHCD4528/ MHCD1628 |
| Ch02 | HMW-MAA (NG2) | AF488 | Veridex | 1 µL |  |
| Ch03 | CD146 | PE | Veridex | 1 µL |  |
| Ch04 | BF | | | | |
| Ch05 | DRAQ5 | | BioStatus | 3.5 µL (of 1:25) | DR50200 |
| Ch06 | (not used) |  |  |  |  |
|  |  |  |  |  |  |
| **Assay #4 – Melanoma (Spiked Cells)** | | | | | |
| **405 nm: 80 mW | 488 nm: 100 mW | 642 nm: OFF | 785 nm: OFF** | | | | | |
| **Channel** | **Antibody** | **Fluor** | **Vendor** | **Volume** | **Part Number** |
| Ch01 | CD45/16 | Pacific Blue | Invitrogen | 1.5 / 1 µL | MHCD4528/ MHCD1628 |
| Ch02 | CD146 | AF488 | Biolegend | 2 µl | 342008 |
| Ch03 | EpCAM | PE | Invitrogen | 2 µL | A15782 |
| Ch04 | BF | | | | |
| Ch05 | DRAQ5 | | BioStatus | 3.5 µL (of 1:25) | DR50200 |
| Ch06 | (not used) |  |  |  |  |
|  |  |  |  |  |  |
| **Assay #5 – Nucleated Cell Deconvolution Panel** | | | | | |
| **405 nm: 80 mW – 488 nm: 100 mW – 642 nm: OFF – 785 nm: OFF** | | | | | |
| **Channel** | **Antibody** | **Fluor** | **Vendor** | **Volume** | **Part Number** |
| Ch01 | CD45/16/34 | Pacific Blue | Invitrogen/Invitrogen/Veridex | 1.5 / 1 / 1 µL | MHCD4528/ MHCD1628Error: Reference source not found |
| Ch02 | Concanavalin A | AF488 | Invitrogen | 1 µL | C11252 |
| Ch03 | Glycophorin A | PE | BioLegend | 1 µL | 349106 |
| Ch04 | BF | | | | |
| Ch05 | DRAQ5 | BioStatus | 3.5 uL (of 1:25) | DR50200 |  |
| Ch06 | CD41 | PE-Cy7 | BioLegend | 1 µL | 303718 |


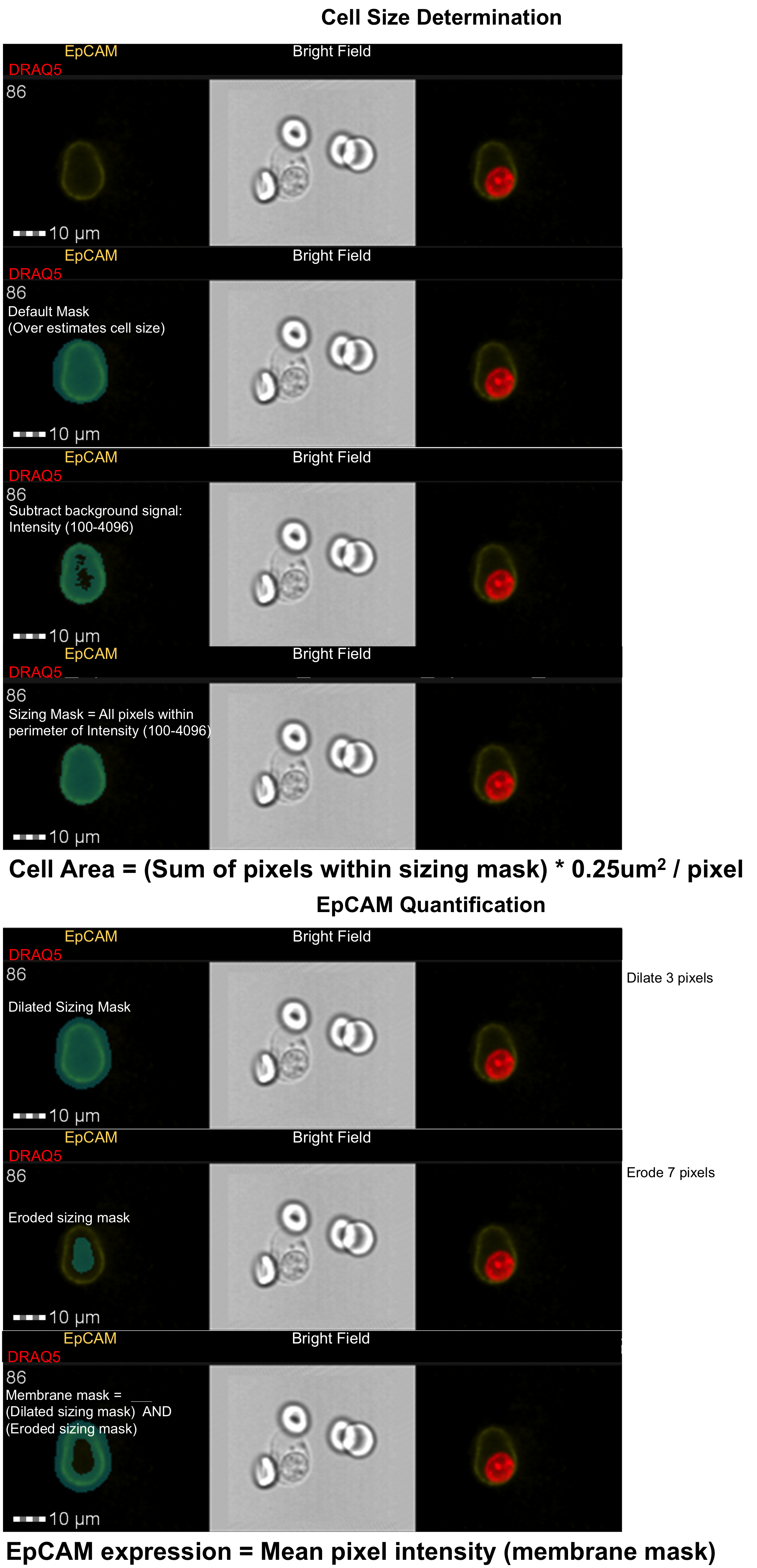


SI Figure 15: Size and EpCAM Expression Measurement Explanation

**Vectra Plating and Staining Methods and Materials:**

For the plating, staining and imaging of cells reported in Figure 3 of the main paper the following procedure was utilized. Several PolyPrep slides (1 for each 1.75mL of isolator product) are marked using a hydrophobic pen in a rectangular outline approximately 2.5cm by 4cm in dimension. The slide is then loaded into a Shandon MegaFunnel with the poly-lysine coating towards the sample chamber, which is then loaded with 2mL of fixed isolator product (2% paraformaldehyde incubated for 10 minutes). A cytospin is performed with the MegaFunnel at 2000RPM for 5 minutes and left for 10 minutes before the slides are removed from the Megafunnel holders. In a staining chamber (blacked out box), the slides are each covered with ~1mL of 1x PBS without overflowing the hydrophobic areas. The staining chamber is then held at a 10 degree angle and the PBS is aspirated gently. The slides are then stored at 4C submerged in 1x PBS in a sealable slide holder until stained. Notes: Do not allow slides to sit without fluid for longer than 30 seconds and in order to minimize evaporation leave the staining chamber closed except when adding or aspirating fluid.

SI Table 7: Staining solutions for multispectral imaging.

|  |  | Breast Cancer Samples | | Prostate Cancer Samples | |
| --- | --- | --- | --- | --- | --- |
| Surface | Clone | Working Concentration (µg/mL) | Dilution | Working Concentration (µg/mL) | Dilution |
| CD16 – AF647 | Mouse monoclonal IgG1, kappa |  | 1:60 |  | 1:60 |
| CD45 – AF647 | Mouse monoclonal IgG1, kappa |  | 1:60 |  | 1:60 |
| CD66b – AF647 | Mouse monoclonal IgG1 | 10 |  | 10 |  |
| EpCAM – AF555 | Mouse monoclonal IgG1 | 5 |  | 5 |  |
| HER2 – AF592  Or  PSA – AF592 | (HER81) Mouse monoclonal IgG1  Or  (D6B1) Rabbit monoclonal Primary and Goat anti-Rabbit IgG Secondary | 5 |  | 5  Or  5 |  |
| Intracellular |  |  |  |  |  |
| CK8/18 (C11) – AF488 | Mouse monoclonal IgG1, kappa | 5 |  | 5 |  |
| CK19-AF488 | Mouse monoclonal IgG2a, lambda | 5 |  | 5 |  |
| DAPI |  |  | 1:500 |  | 1:500 |

Slides are first blocked for 30 minutes with 3% Bovine Serum Albumin and 2% goat serum in 1x PBS. Surface staining solution is added and incubated at room temperature for 1 hour protected from light before washing 3 times with 1x PBS. Next, the sample is washed once with 0.3% Tween20 in 1x PBS then incubated in the same solution for 15 minutes to permeablize the cells. The slides are then washed 3 times with 1x PBS followed by a wash with 0.3% Tween20 in 1x PBS after which the intracellular staining solution is added and incubated for 1 hour at room temperature protected from light. The slides are washed with 0.3% Tween20 in 1x PBS twice then 1x PBS twice and finally 2 drops of mounting medium is placed on the sample. The slides are kept flat for 24 hours or until the mounting medium is dry. Slides should be stored at 4C and protected from light.

All samples are imaged using a Vectra Multispectral Imaging platform.

**Cell Culture Details:**

For each of the cell lines below we tried to maintain a passaging schedule where we split twice a week on Monday and Friday. We would subsequently change the media on Wednesday.

| **PC3-9 - MGH** | |
| --- | --- |
| Experiment Purpose: | Used for cell spiking and microfluidic processing. |
| Medium: | RPMI-1640, HEPES, L-Glutamine  i.e. Cat # A10491 (Invitrogen)  10% Fetal Bovine Serum  1% Pen Strep |
| Subcultivation Ratio: | 1:5 to 1:20 |
| Cell Dissociation Solution: | 0.05% (w/v) Trypsin |
| Trypsin Volume: | 3mL |
| Trypsin Incubation: | 5 min at 37^o^C |
| Medium Renewal: | Every 3 days |
| Notes: | Do not hit or shake flask while waiting for cells to detach. |

| **SKBR** | |
| --- | --- |
| Experiment Purpose: | Used for cell spiking and microfluidic processing. |
| Medium | RPMI-1640, HEPES, L-Glutamine  i.e. Cat # A10491 (Invitrogen)  10% Fetal Bovine Serum  1% Pen Strep |
| Subcultivation Ratio: | 1:2 to 1:10 |
| Cell Dissociation Solution: | *0.05% (w/v) Trypsin* |
| Trypsin Volume: | 3 mL |
| Trypsin Incubation: | 5 minutes at 37^o^C |
| Medium Renewal: | Every 3 days |
| Notes: | Prone to clumping. Do not hit or shake flask while waiting for cells to detach. |

| **MB231** | | |
| --- | --- | --- |
| Experiment Purpose: | | Used for cell spiking and microfluidic processing. |
| Medium: | | DMEM, High Glucose  i.e. Cat # 11965-092 (Invitrogen)  10% Fetal Bovine Serum  1% Pen Strep |
| Subcultivation Ratio: | | 1:5 to 1:15 |
| Cell Dissociation Solution: | | 0.05% (w/v) Trypsin |
| Trypsin Volume:: | 3mL | |
| Trypsin Incubation: | | 5 min at 37^o^C |
| Medium Renewal: | | Every 3 days |
| Notes: | | Do not hit or shake flask while waiting for cells to detach. |

| **SK-Mel-28 - MGH** | |
| --- | --- |
| Experiment Purpose: | Used for cell spiking and microfluidic processing. |
| Medium: | RPMI-1640, HEPES, L-Glutamine  10% Fetal Bovine Serum  1% Pen Strep |
| Subcultivation Ratio: | 1:3 to 1:8 |
| Cell Dissociation Solution: | 0.05% (w/v) Trypsin |
| Trypsin Volume: | 3mL |
| Trypsin Incubation: | 5 min at 37^o^C |
| Medium Renewal: | Every 2-3 days |
| Notes: | Do not hit or shake flask while waiting for cells to detach. |

| **H1975** | | |
| --- | --- | --- |
| Experiment Purpose: | | Used for cell spiking and microfluidic processing. |
| Medium: | | RPMI-1640, HEPES, L-Glutamine  10% Fetal Bovine Serum  1% Pen Strep |
| Subcultivation Ratio: | | 1:8 |
| Cell Dissociation Solution: | | 0.05% (w/v) Trypsin |
| Trypsin Volume:: | 3mL | |
| Trypsin Incubation: | | 5 min at 37^o^C |
| Medium Renewal: | | Every 2-3 days |
| Notes: | | Do not hit or shake flask while waiting for cells to detach. |

For all cell lines:

- Use 25cm^2^ cell culture flasks (Becton, Dickinson, C/N 353108)
- Atmosphere: air, 95%; carbon dioxide (CO2), 5%
- Temperature: 37.0°C
- Suggested Fetal Bovine Serum (Invitrogen C/N 16000-044)
- Suggested Trypsin (Invitrogen C/N 25300054)

**REFERENCES**

1 Rapp, B.E. Microfluidics: Modeling, Mechanics and Mathematics. pp 359-362. (Elsevier, Cambridge, MA, USA, 2017).
